# Multiscale Biomimetic Evaporators Based on Liquid Metal/Polyacrylonitrile Composite Fibers for Highly Efficient Solar Steam Generation

**DOI:** 10.1007/s40820-025-01661-z

**Published:** 2025-02-05

**Authors:** Yuxuan Sun, Dan Liu, Fei Zhang, Xiaobo Gao, Jie Xue, Qingbin Zheng

**Affiliations:** School of Science and Engineering, The Chinese University of Hong Kong, Shenzhen, 518172 Guangdong People’s Republic of China

**Keywords:** Liquid metal, Polyacrylonitrile, Composite fibers, Solar steam generation, Seawater desalination

## Abstract

Solar steam generation (SSG) offers a cost-effective solution for producing clean water by utilizing solar energy. However, integrating effective thermal management and water transportation to develop high-efficiency solar evaporators remains a significant challenge. Here, inspired by the hierarchical structure of the stem of bird of paradise, a three-dimensional multiscale liquid metal/polyacrylonitrile (LM/PAN) evaporator is fabricated by assembling LM/PAN fibers. The strong localized surface plasmon resonance of LM particles and porous structure of LM/PAN fibers with interconnected channels lead to efficient light absorption up to 90.9%. Consequently, the multiscale biomimetic LM/PAN evaporator achieves an outstanding water evaporation rate of 2.66 kg m^−2^ h^−1^ with a solar energy efficiency of 96.5% under one sun irradiation and an exceptional water rate of 2.58 kg m^−2^ h^−1^ in brine. Additionally, the LM/PAN evaporator demonstrates a superior purification performance for seawater, with the concentration of Na^+^, Mg^2+^, K^+^ and Ca^2+^ in real seawater dramatically decreased by three orders to less than 7 mg L^−1^ after desalination under light irradiation. The multiscale LM/PAN evaporator with hierarchical structure regulates the water transportation as well as thermal management for highly effective solar-driven evaporation, providing valuable insight into the structural design principles for advanced SSG systems.

**Supplementary Information:**

The online version contains supplementary material available at 10.1007/s40820-025-01661-z.

## Highlights

- A three-dimensional multiscale liquid metal/polyacrylonitrile evaporator is fabricated through wet spinning and assembly.
- The evaporator exhibits an outstanding water evaporation rate of 2.66 kg m^−2^ h^−1^ with a solar energy efficiency of 96.5% under one sun irradiation.
- The evaporator demonstrates a superior purification performance for seawater and sewage under light irradiation.

**Supplementary Information:**

The online version contains supplementary material available at 10.1007/s40820-025-01661-z.

## Graphical abstract

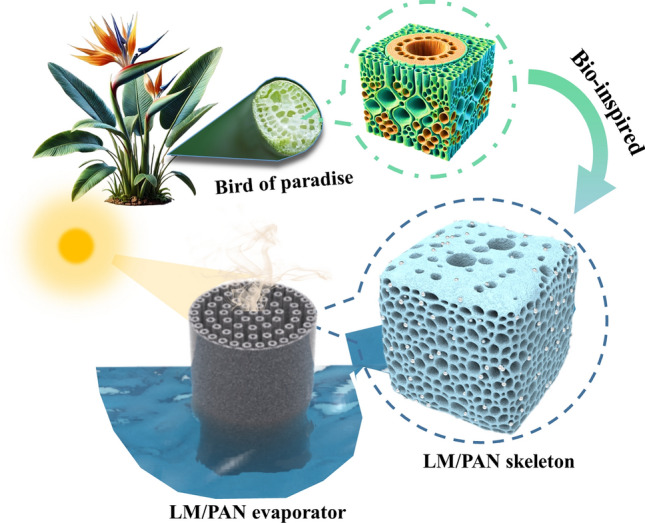

## Introduction

With the boosting population growth and severe environmental pollution, global freshwater scarcity becomes an essential issue confronting more than three billion people [1–3]. Consequently, an urgent demand arises for the development of facile and effective water purification and desalination technologies [4–6]. To date, a variety of technologies, including thermal distillation, electrodialysis and reverse osmosis, are used for water purification and desalination [7]. However, most of these technologies rely on non-renewable fossil fuels, leading to elevated emissions [8]. In contrast, solar steam generation (SSG), which utilizes eco-friendly solar energy to generate potable water, has garnered considerable attention [9–12].

An ideal SSG system features strong light absorption, effective thermal management, as well as prompted water transportation [13, 14]. The light absorption is greatly determined by the properties and microstructure of photothermal material [15]. Thus, various photothermal materials have been developed, including plasmonic particles [16–18], semiconductors [19, 20], carbonaceous materials [21–24] and polymers [25–27]. Eutectic gallium/indium, a near-room-temperature liquid metal (LM), exhibits outstanding plasmonic effect similar to gold [28–30] and silver particles [31], enabling LM particles as potential photothermal materials utilized in SSG system [32–34]. Meanwhile, LM particles demonstrate significant potential for high-efficiency thermal management due to the high latent heat, heat flux density as well as lower density compared to traditional metals [35–37]. Compared with carbon nanomaterials such as carbon nanotube and graphene, which suffer from significant heat loss during conduction due to their high thermal conductivity [38, 39], LM particles process a relatively low thermal conductivity (26.6 W m^−1^ K^−1^) [40]. More importantly, LM particle triggers a better isolation when integrated into substrate, leading to the reduced heat loss [41].

Additionally, LM particles exhibit superior hydrophilicity compared to gold and silver particles due to the easy formation of a thin oxide film on their surface [42, 43]. There are three kinds of water clusters, including free water (FW), intermediate water (IW) and bound water (BW), according to the different interactions between water molecules [44]. Due to weaker interaction with adjacent water molecules, the evaporation rate of IW is significantly higher than that of FW [45, 46]. By incorporating LM particles with various hydrophilic materials, such as polyvinyl alcohol [47], cellulose [48] and chitosan [49], the interactions between water and evaporator were enhanced, leading to the increase of IW and the decrease in the evaporation enthalpy [50–53]. Consequently, it can be inferred that LM particle can achieve effective photothermal conversion, low heat loss and suitable water supply ability, which is beneficial to enhance the SSG performance [54]. However, the fluidic nature of LM leads to the weak compatibility with polymers, posing challenges for preparing solid LM–polymer composites [55].

The structure of solar steam generator is another essential factor that significantly affects the thermal management and water transportation [56]. Typically, the water evaporation rate of conventional 2D evaporator is restricted with a theoretical limit of 1.6 kg m^−2^ h^−1^ under one sun irradiation [57]. The unsatisfied SSG performance is attributed to the significant heat loss due to the direct contact between photothermal materials and bulk water [58]. In contrast, evaporators with 3D architecture can effectively improve the solar evaporation rate and energy conversion efficiency. The increased overall evaporation surface area and lower surface evaporation temperature provide the 3D evaporator with reducing thermal radiation and convection energy losses [59, 60]. To ensure continuous steam generation, it is also important to improve the quality of water delivery. 3D interconnected porous structure was proved to be effective for water transportation because the abundant and open pores guarantee the continuous water transportation from the bottom reservoir to the upper evaporation surface using sufficient capillary effect as a driving force [61]. However, the infiltration of water into open pores invariably gives rise to direct contact between water and photothermal materials as well as increased thermal conductivity of evaporator, resulting in higher heat loss to the bulk water through conduction and further diminishing the heat localization effect [57]. A potential strategy to minimizing the heat loss is to separate the water transportation from the water evaporation through structure engineering. The pores for the water transportation are designed to remain open, creating a sufficient and continuous channel. Meanwhile, the photothermal materials are separated from the bulk water by a thermal insulator, ensuring efficient photothermal conversion and thermal management. Until now, challenges persist in fabricating a 3D SSG system that combines an exceptional solar vapor evaporation rate with high energy conversion efficiency. Consequently, effectively separating the water transportation channels from the photothermal conversion areas has emerged as a pivotal strategy for developing a highly efficient three-dimensional SSG system.

Natural vascular plants powered by sunlight utilize differences in osmotic pressure, transpiration as well as guttation to produce tons of clean water. As a typical kind of vascular plants, bird of paradise with vertically aligned channels in the stems demonstrate outstanding water transportation for ensuring survival in hot environments [62, 63]. Specifically, the tracheid and vessel tissue in the stems are interconnected to form a continuous water-conducting micron channel system extending throughout the plant [64]. Here, inspired by the stem structure of the bird of paradise with effective water transport ability, we propose an efficient design strategy to produce multiscale biomimetic solar evaporators based on assembled liquid metal/polyacrylonitrile (LM/PAN) fibers via wet spinning that features a LM particle decorated sponge-like PAN microstructure. The assembled bionic LM/PAN evaporator consisted of vertically aligned LM/PAN fibers with hierarchical porous structure. During solar evaporation, LM particles served as an effective photothermal material to improve the conversion efficiency. Meanwhile, coupled with hydrophilic characteristic, the high porosity of tailored 3D PAN network provided the LM/PAN evaporator with efficient water transport pathway as well as thermal insulation layer for isolating the photothermal conversion areas, thus effectively minimizing the heat loss. The exceptional performance of LM/PAN evaporator on seawater desalination and water purification was also evaluated. Additionally, an LM/PAN evaporator-based water purification prototype was developed to demonstrate the superior SSG performance, revealing the potential of LM/PAN evaporator for scalable manufacturing in future practical applications.

## Experimental Section

### Materials

Liquid metal (containing 75% Ga and 25% In) was purchased from Dongguan Hua Titanium Material Technology Co. Ltd. Polyacrylonitrile (PAN, molecular weight 150,000), sodium chloride (NaCl, AR, 99.5%), N, N-dimethylformamide (DMF) and potassium carbonate (K_2_CO_3_) used in dark experiment were bought from MACKLIN, Co., Ltd. Methylene blue was obtained from Hunan BKMAM Holding Co. Ltd. Nitric acid (HNO_3_) was provided by Guangzhou Chemical Reagent Factory.

### Preparation of Biomimetic LM/PAN Evaporator

#### Preparation of LM/PAN Fibers

The purchased PAN powder was initially dispersed into the DMF with a weight ratio of 1:9. The resulting solution was magnetic stirred at 1000 rpm for 6 h under ambient temperature until PAN was completely dissolved into the DMF. LM was then added to the PAN solution, and the mass ratio of LM to PAN was tailored to 0, 1:1, 2:2 and 3:1. The obtained PAN/LM solution was magnetic stirred, followed by 15-min sonication for to obtain a uniform PAN/LM spinning dope.

#### Preparation of Biomimetic 3D LM/PAN Evaporator

A laboratory-scale, single-filament wet spinning method was used to fabricate the LM/PAN fibers. The LM/PAN dope was extruded through a 20-mL syringe with a 1-mm-diameter needle by a syringe pump at a speed of 300 mL h^−1^. Subsequently, the spun fibers were solidified in a water coagulation bath. The prepared fibers were then cut to uniform sizes and bundled together using two wires on each side to obtain a cylinder with all fibers vertically aligned. To control the diameter of cylinder, each cylinder consisted of 64 LM/PAN fibers. Afterward, 10 mL as-prepared LM/PAN dope was coated on the surface of the LM/PAN fibers and solidified in a water bath. The resulting wet LM/PAN bundle was frozen at 0 °C, following by cutting off the ends bundled with wires. Finally, the LM/PAN bundle was freeze-dried to obtain the LM/PAN evaporator with a fixed length of 2.5 cm. Pure PAN fibers and LM/PAN fibers fabricated by the weight ratio of LM to PAN at 1:1, 2:1, 3:1 are hereafter denoted as PAN evaporator, LM/PAN_11_, LM/PAN_21_ and LM/PAN_31_ evaporator, respectively. Besides, to investigate the effect of the diameter of LM/PAN fibers, different diameters of needle were used. The LM/PAN fibers were fabricated using needles with diameter of 0.84 and 1.30 mm while the weight ratio of LM to PAN in the spinning dope maintained at 2:1. Consequently, all LM/PAN evaporators solar steam generators have a comparable diameter around 12 mm and a length of 2.5 cm.

### Measurement of Water Absorption Capability

The saturated water content was represented by the weights of absorbed water in PAN evaporator and LM/PAN evaporators during the water absorption process sample per gram of corresponding dried PAN evaporator and LM/PAN evaporators, respectively. PAN evaporator and LM/PAN evaporators were vertically placed in a container with pure water. The weight change of the samples was recorded by an electronic balance every 20 s for 600 s. Before measuring the mass, PAN evaporator and LM/PAN evaporators were wiped by bibulous paper. Due to the strong capillary of bibulous paper, most of the surface attached water was removed.

### Measurement of SSG Performance

Polystyrene (PS) foam was first cut into cylinders with a diameter of 30 mm and a height of 25 mm. A circular hole with a diameter of 12 mm was then cut in the center of the cylinder. Subsequently, the PAN evaporator and LM/PAN evaporator were fixed in the hole and the foam was put into a glass container with a diameter of 30 mm**.** After exhibiting the apparatus for SSG, a solar simulation (SUN 3000, ABET) was used to simulate the solar radiation. Meanwhile, a power meter (DT-1307, CEM) was employed to detect and maintain the solar radiation intensity at 1 kW m^−2^. Consequently, the mass of water loss was recorded every 10 min by an electronic balance.

### Calculation of Solar Energy Conversion Efficiency

The solar energy conversion efficiency (*η*) for SSG was defined as [13, 65, 66]:

1 $$\eta = \frac{{m_{se} (\Delta H_{eq} + \Delta H_{s} )}}{{P_{o} C_{opt} }}$$

2 $$\Delta H_{s} = C_{w} \Delta T$$

where m_se_ represents the solar contributed water evaporation rate. Typically, m_se_ is calculated as difference between total evaporation rate under solar irradiation and evaporation rate in dark evaporation experiment. ${C}_{w}$ is the specific heat of water (4.18 J g^−1^ K^−1^), $\Delta T$ is the absorbing surface temperature difference, C_opt_ is the solar power absorption coefficient on the evaporation surface and P_0_ is the solar power intensity (1 kW m^−2^ for 1 sun).

### Molecular Dynamics (MD) Simulations

MD simulations were conducted to investigate the underlying reduction mechanism of evaporation enthalpy in LM/PAN evaporators. All MD simulations were performed using Materials Studio 2023 (BIOVIA) software. Water, PAN and liquid metal layers were all constructed using Amorphous Cell modulus. Specifically, for water layer, 500 water molecules were packed into a box with a diameter of 25Å×25 Å×23 Å at a preset density of 1 g cm^−3^. For PAN layer, 15 PAN chains that each consisted of 10 monomers were packed into a box with a diameter of 25 Å×25 Å×21 Å at a preset density of 1.14 g cm^−3^. To construct the LM/PAN layer, the liquid metal model with a diameter of 10.8 Å×7.2 Å×3.6 Å was supercell from the unit cell of liquid metal, which dimension is 3.6 Å ×3.6 Å ×3.6 Å. Subsequently, the model was packed with 15 PAN chains to ensure the weight ratio of LM to PAN was 2. The as-constructed water, PAN and LM/PAN layer were first optimized using geometric optimization task and relaxed under NVT (constant-temperature, constant-volume) ensemble at 313 K for 200 ps with a step length of 1 fs.

To construct the water–water, water–PAN and water–LM/PAN evaporation simulation system, the water layer was assigned to the surface of the other same water layer, PAN layer and LM/PAN layer with a vacuum gap of 2 Å to prevent molecular overlap. Meanwhile, a 150 Å vacuum slab was add to the upper surface of the water layer to construct the non-periodic condition. After construct three evaporation models, geometric optimization was firstly carried out to minimize the energy of the system. Molecular dynamics simulation under NVT ensemble was used to simulate the evaporation processing at 313 K for 500 ps with a step length of 1 fs. Universal force field was employed to describe the intermolecular interactions, and the cutoff distance was set as 12.5 Å. Nose thermostat was employed to perform constant-temperature dynamics in all molecular dynamic simulations. Finally, the amount of hydrogen bonds during water molecules evaporation in all systems as the function of time was collected using scripts.

### Characterization

Scanning electron microscope (SEM, MAIA 3, TESCAN, Czech) was used to observe the microstructure of LM/PAN evaporator. The typical functional groups were verified by Fourier transform infrared spectroscopy (FTIR, Spectrum Two, PerkinElmer, USA). X-ray diffractometer (XRD, SmartLab, Rigaku, Japan) was performed to study the crystal structure evolution in LM/PAN evaporator. The elemental compositions were characterized by X-ray photoelectron spectroscopy (XPS, Axis Supra + , Kratos, Japan). Ultraviolet–visible–near-infrared spectrophotometer (UV–Vis–NIR, UV-3600, Shimadzu, Japan) was conducted to estimate the absorption of LM/PAN evaporators and methylene blue solution. Optical contact angle instrument (OCA, 15EC, Dataphysics, Germany) was used to measure the wettability. Differential scanning calorimetric measurement (DSC, DSC2500, TA Instruments, USA) was used to obtain water vaporization enthalpy. The infrared (IR) photography was observed with the infrared camera (226 s, FOTRIC, China). The metal ion concentration was estimated using inductively couple plasma–optical emission spectrometer (ICP-OES, Avio 220 Max, PerkinElmer, USA). Raman spectrometer (Renishaw, inVia, UK) was used to analyze the hydration state of water.

## Results and Discussion

### Design and Fabrication of Multiscale Biomimetic Evaporator Structure

**Fig. 1:**
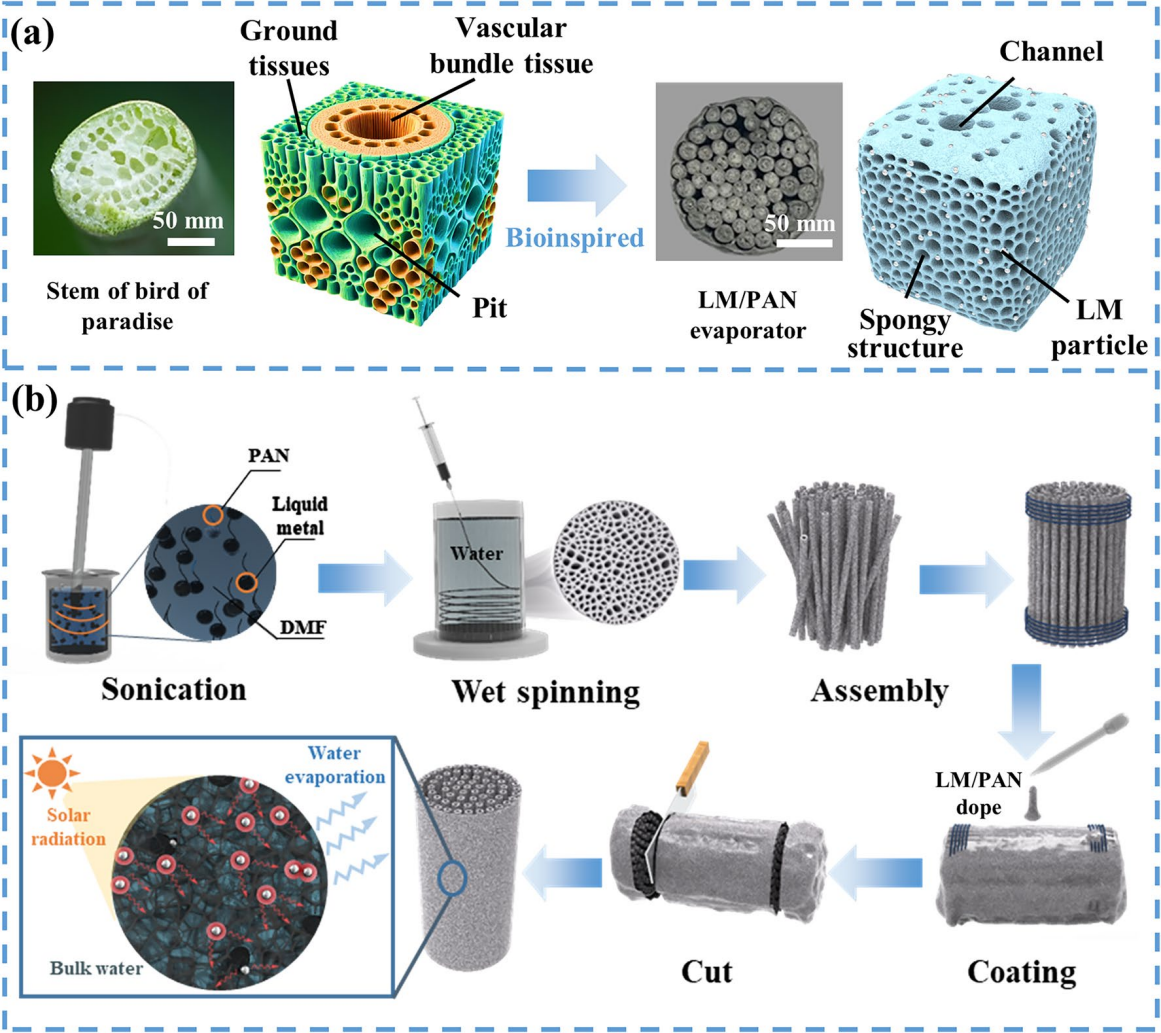
Design of biomimetic multiscale LM/PAN evaporator. **a** Optical images and schematic diagrams of the stem of bird of paradise and LM/PAN evaporator. **b** Schematic diagram of the fabrication of LM/PAN evaporator

As shown in Fig. 1a, as a vascular plant with a crucial role in the ecosystem, the stem of bird of paradise evolves an efficient hydraulic system with porous cross-sectional area to ensure effective water transportation during the process of transpiration. Moreover, the stem showed a microstructure configuration that circular ground tissues were densely arranged into a sponge-like structure, which was responsible for photosynthesis as well as storage of water (Fig. S1a). The vascular bundle tissue was located within the ground tissues (Fig. S1b). Specifically, in vascular bundle tissue, the tracheid elements with diameters of 20–40 μm were distributed around the vessel elements, constructing an effective channel to pump water from the roots. Moreover, the distinctive microstructures of cell walls, such as pits and plasmodesmata, enabled the flow of water from one element to another, forming an effective water transport channel and linking water uptake in roots with photosynthesis in ground tissues. Inspired by the special microstructure in bird of paradise stem, the biomimetic LM/PAN evaporator featuring hierarchical structure along with high porosity was designed and fabricated (Fig. 1a). As the basic unit of LM/PAN evaporator, LM/PAN fibers were prepared by mixing the LM particle and PAN followed by wet spinning as shown in Fig. 1b. In contrast to previous works on wet spinning of LM, which primarily focused on developing core–sheath fibers for soft electronics through coaxial wet spinning [67, 68], LM was first mixed with PAN in DMF solution and then fabricated into fibers via wet spinning in a water bath. Due to the phase separation of PAN from DMF solution, LM/PAN fibers comprised hierarchical micro-/nanostructured channels through the entire fibers. The unique microstructure synergistically imparted the SSG system with requisite efficient water transportation and steam vapor pathway, similar to the vascular bundle tissue of bird of paradise stem. Beneath the surface of channels inside the LM/PAN fibers, the phase separation created spongy structure made up of a composite containing PAN along with LM particles. The spongy structure enhanced the reflection and refraction of the incident sunlight as well as the effective water evaporation area. The strong plasmonic effect of LM particle contributed effective conversion between solar light and thermal energy. Moreover, as the primary skeleton of LM/PAN fibers, PAN featured low thermal conductivity and hydrophilicity. Thus, the LM/PAN fibers with porous sponge-like structure were expected to reduce the heat loss, promoting efficient thermal management of heat energy converted by LM particles. Finally, LM/PAN fibers were coated with LM/PAN dope and vertically aligned to assemble LM/PAN evaporator, which mimicked the micron channel found in the bird of paradise stem, promoting the water pumping and enhancing the structural stability during evaporation. The key to high-performance SSG in LM/PAN evaporator not only relied on the efficient water transport channel, but also spatially separated water evaporation area and water transport channel into different regions to reduce heat loss.

### Characterization of LM/PAN Fibers

**Fig. 2:**
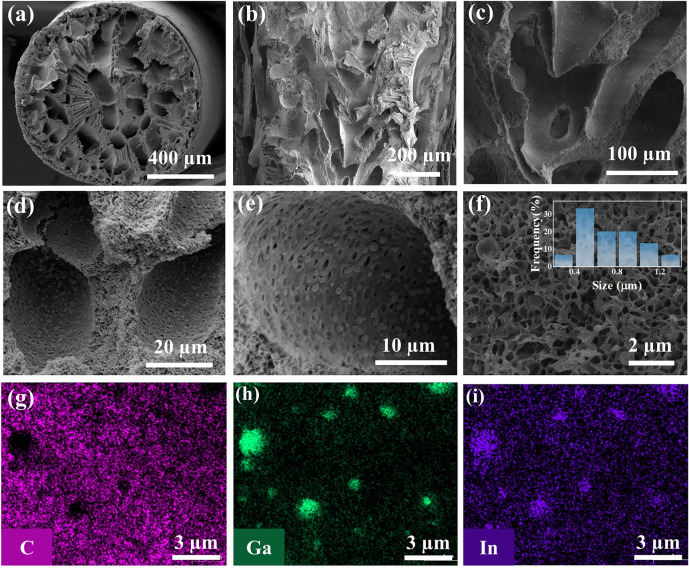
Microstructure of LM/PAN fibers. SEM images of **a** cross section of LM/PAN fibers, **b** longitudinal section of LM/PAN fibers, **c** micron channels in **b**, **d-e** porous structures on the surface of micron channel. **f** SEM image of cross section of the channel wall and **g**–**i** the corresponding elemental mapping images

As shown in Fig. 2a, the diameter of LM/PAN fibers was around 1mm and LM/PAN fibers exhibited porous structure with long channels. For the longitudinal section (Fig. 2b), networks of intersected micron channels with a width ranging from ~ 10 to 50 µm were observed throughout LM/PAN fibers, forming an effective water transportation structure similar with tracheid and vessel elements in bird of paradise. The high-magnification SEM image (Fig. 2c) indicates that the channel wall exhibited a rough surface texture, which was beneficial to increase the traversing distance of incident light as well as promoting light absorption. The hierarchical structure of LM/PAN fibers was further confirmed from the microstructure of the channel wall (Fig. 2d). Specifically, densely packed pores with size around 1 µm were uniformly distributed on the surface of the channel wall (Fig. 2e). Meanwhile, the dense array of micron pores formed a porous spongy structure inside the channel wall. The LM particles with average diameter of 0.72 µm were decorated on the inner channel wall. As shown in Fig. 2f–i, carbon element was uniformly distributed on the PAN skeleton. In addition, a uniform distribution of Ga and In elements was also observed on the spherical structure, verifying the decoration of LM particle on the PAN skeleton.

**Fig. 3:**
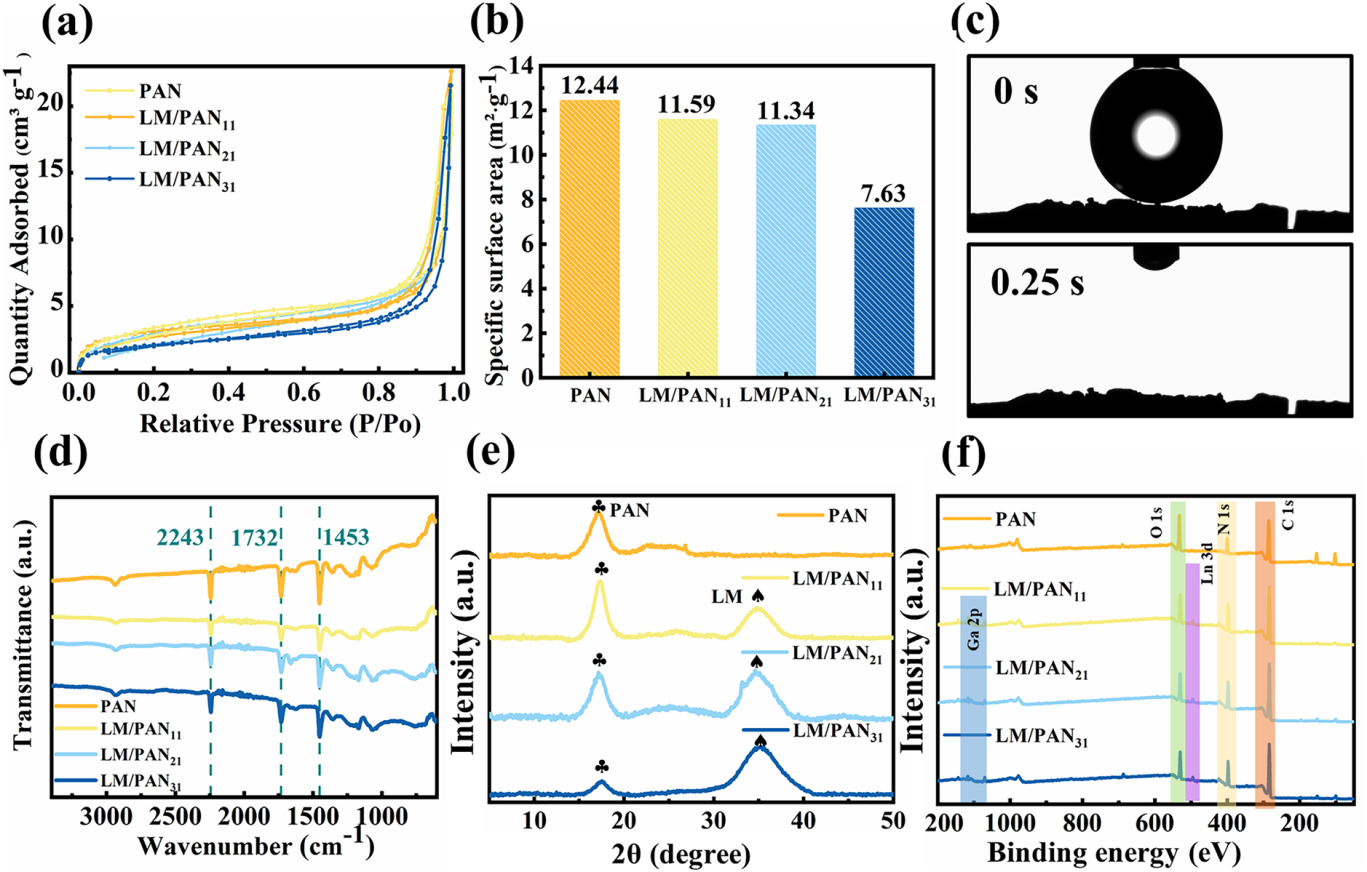
Characterization of the PAN fibers and LM/PAN fibers. **a** N_2_ adsorption and desorption curve. **b** Specific surface area of PAN fibers and LM/PAN fibers. **c** Water contact angle of LM/PAN_21_ fibers. **d** FTIR spectra, **e** XRD patterns, **f** XPS spectrum of PAN fibers and LM/PAN fibers

More importantly, the microstructure of LM/PAN fibers could be controlled by changing the weight ratio between PAN and LM during the fabrication process. Specifically, LM/PAN fibers with various weight ratios of LM to PAN (1:1, 2:1, 3:1) were fabricated, namely LM/PAN_11_, LM/PAN_21_ and LM/PAN_31_ fibers respectively. As shown in Fig. S2, both PAN fibers and LM/PAN_11_ fibers featured a porous channel surface along with spongy structure (Fig. S2). As the LM content increasing, pores on the channel wall were occupied by the LM particles. In LM/PAN_31_ fibers (Fig. S2j-l), LM particles were densely distributed on the channel surface and obscured the initial porous PAN structure, which was unfavorable to water transportation. The porosity change was further demonstrated by the evolution of specific surface area as the LM content increasing. As shown in Fig. 3a, b, the specific surface area reduced from 12.44 m^2^ g^−1^ in PAN fibers to 7.63 m^2^ g^−1^ in LM/PAN_31_ fibers, confirming that the pores on the channel wall were occupied by increasing LM particles. As seen from Figs. 3c and S3, water droplet was absorbed within 0.25 s from the top surface due to the hydrophilicity of PAN and LM particles and the strong capillary force of vertical aligned LM/PAN fibers. Therefore, the hydrophilicity of LM/PAN evaporators were negligibly affected as the specific surface area changed.

To further validate the integration of LM and PAN in LM/PAN fibers, FTIR, XRD and XPS of LM/PAN fibers were also conducted. The FTIR spectrum of PAN fibers and LM/PAN fibers (Fig. 3d) show characteristic peaks of PAN at 2243 and 1453 cm^−1^ [69], corresponding to the nitrile group (C≡N) and the C-H bond. respectively Additionally, the peak appeared at 1732 cm^−1^ was attributed to the stretching vibration of the carbonyl group (C = O) in DMF [70]. Therefore, the PAN remained unchanged after the introduction of LM particles. The XRD patterns of PAN fibers and LM/PAN fibers (Fig. 3e) show a diffraction peak at 2θ = 16.1°, corresponding to the (100) plane of PAN [71]. Compared with the XRD patterns of PAN fibers, the characteristic peak of LM at 34.5° was observed in LM/PAN fibers [33]. Furthermore, the peak intensity of LM increased from LM/PAN_11_ fibers to LM/PAN_31_ fibers, indicating the increase in the LM content. The element composition of PAN fibers and LM/PAN fibers were investigated by XPS spectra (Fig. 3f). Compared with the XPS spectrum of PAN, the emissions of Ga 2*p* and In 3*d* were observed at high (1116 − 1145 eV) binding energy and low regions (443 eV) in LM/PAN fibers [72]. Additionally, the high-resolution Ga 2*p* XPS spectrum of LM/PAN fibers showed two peaks at 1116 and 1143 eV (Fig. S4), which was assigned to the elemental gallium in LM and Ga_2_O_3_ caused by surface oxidation, respectively [35, 43].

### Characterization of LM/PAN Evaporators

**Fig. 4:**
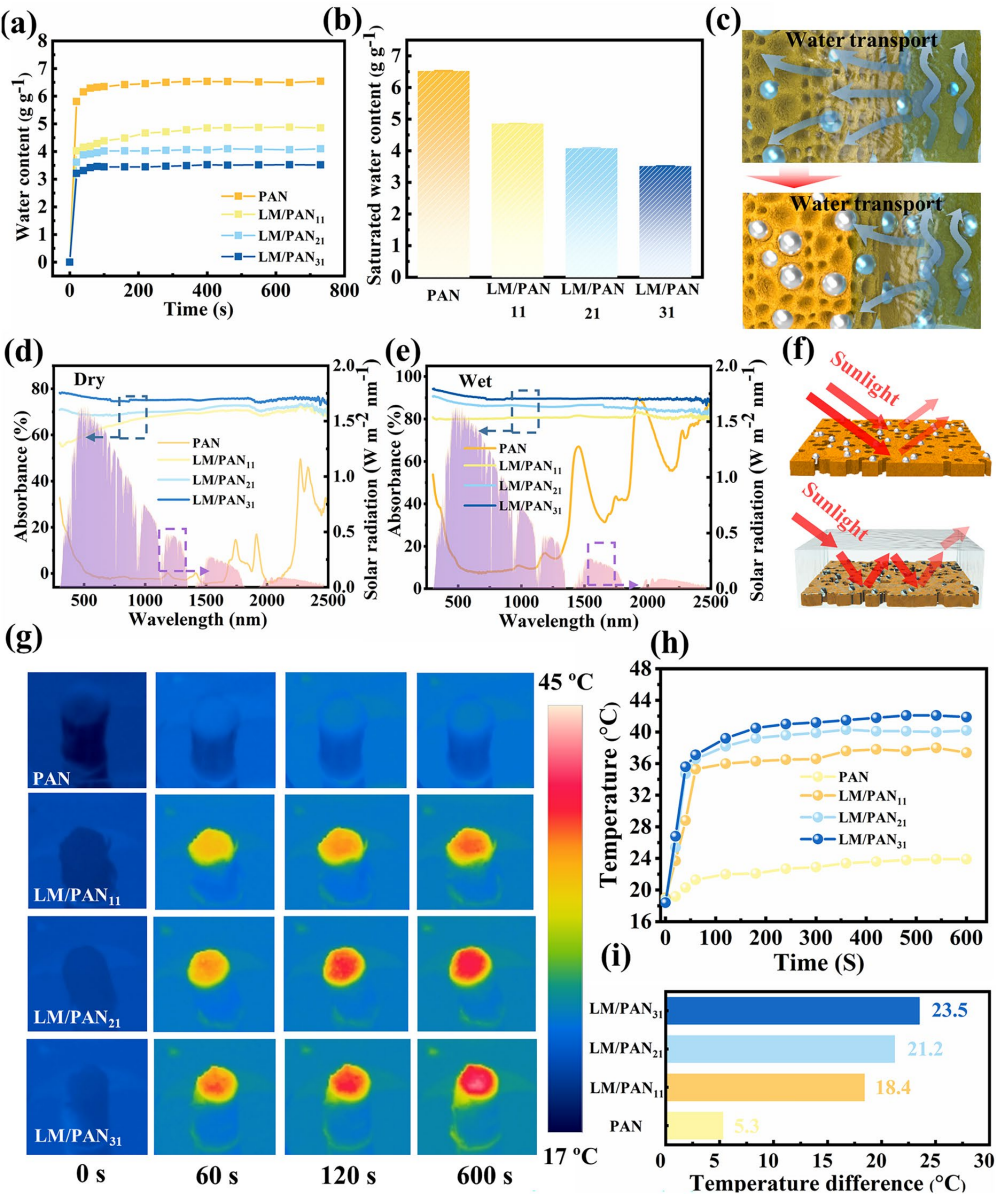
Water transportation and photothermal performance of PAN evaporator and LM/PAN evaporators. **a** Water content variation over time and **b** relative saturated water content in PAN evaporator and LM/PAN evaporators. **c** Schematic of water transportation in LM/PAN evaporators. The UV–Vis–NIR spectra of **d** dry and **e** PAN evaporator and LM/PAN evaporators. The background in **d** and **e** is the normalized spectral solar irradiation of air mass 1.5 global (AM 1.5 G). **f** Schematic illustration of sunlight path on the surface of LM/PAN evaporator before and after water wetting. **g** IR image. **h** Top surface temperature evolution over time and **i** temperature difference between 0 and 600 s in PAN evaporator and LM/PAN evaporators

To investigate the effect of LM particles, the PAN fibers and LM/PAN fibers were assembled to PAN evaporator and LM/PAN evaporators for SSG (Fig. S5), respectively. Due to the large-scale production of wet spinning and facile bundle strategies, LM/PAN fibers can be easily assembled and consist of evaporators with various configurations (Fig. S6), enabling scalability and adaptability for different application scenarios. In this study, the evaporators were scaled to a cylinder configuration with a diameter of 12 mm and a height of 25 mm. The water absorbing capability was firstly evaluated by measuring the water content of PAN and LM/PAN evaporators (Fig. 4a, b). The water content of PAN evaporator reached 5.8 g g^−1^ at 20 s, which is 1.8 times of LM/PAN_31_ (3.2 g g^−1^) evaporator. When the water content was stable, PAN evaporator exhibited a high saturated water content of 6.5 g g^−1^, followed by the LM/PAN_11_ (4.9 g g^−1^), LM/PAN_21_ (4.1 g g^−1^) and LM/PAN_31_ (3.5 g g^−1^). The decreasing water absorption capability was attributed to the reduced porosity of LM/PAN fibers due to the increase in the LM content. As illustrated in Fig. 4c, as the LM content increased, the pore on channel wall and the sponge-like structure were partially filled with LM particles, which was confirmed by the SEM images (Fig. S2) along with the decreasing specific surface area (Fig. 3b), thereby leading to the reduction in the water transportation. The photothermal performance of PAN evaporator and LM/PAN evaporators were then analyzed through investigating both light absorption and light-to-heat conversion abilities. As shown in Fig. 4d, the average absorption of PAN evaporator was only 5.1% within 300–2500 nm. Compared with PAN evaporator, the light absorption of LM/PAN evaporators were significantly enhanced with the addition of LM particles due to the excellent localized surface plasmon effect of LM particles. As the LM content increased, the average absorption reached 75.6% for LM/PAN_31_ evaporator. In addition, the light absorption of PAN evaporator and LM/PAN evaporators was further enhanced after absorbing water (Fig. 4e**)**. Specifically, LM/PAN_11_, LM/PAN_21_ and LM/PAN_31_ evaporator exhibited an outstanding average light absorption of 80.8%, 85.8% and 90.9% from 300 to 2500 nm after absorbing water, respectively. The enhancement was attributed to the decrease in light reflectivity because the interface changed from air–LM/PAN evaporator to the air–water–LM/PAN evaporator interface (Figs. 4f and S7) [73]. To evaluate the light-to-heat conversion abilities, the surface temperatures of PAN evaporator and LM/PAN evaporators were monitored under one sun irradiation using an infrared camera. As shown in Fig. 4g, h, the surface temperature of LM/PAN evaporators increased quickly to over 34 °C in the first 50 s in contrast to that of PAN evaporator (22 °C), and then, the temperatures slowly increased in 50-600 s. Consequently, LM/PAN_31_ evaporator exhibited a significant temperature rise of 23.5 °C. The surface temperature of LM/PAN_21_ evaporator difference showed a slightly lower increase (21.2 °C), followed by LM/PAN_11_ evaporator (18.4 °C). PAN evaporator showed a lowest temperature difference of 5.3 °C (Fig. 4g).

### Solar Steam Generation of LM/PAN Evaporators

**Fig. 5:**
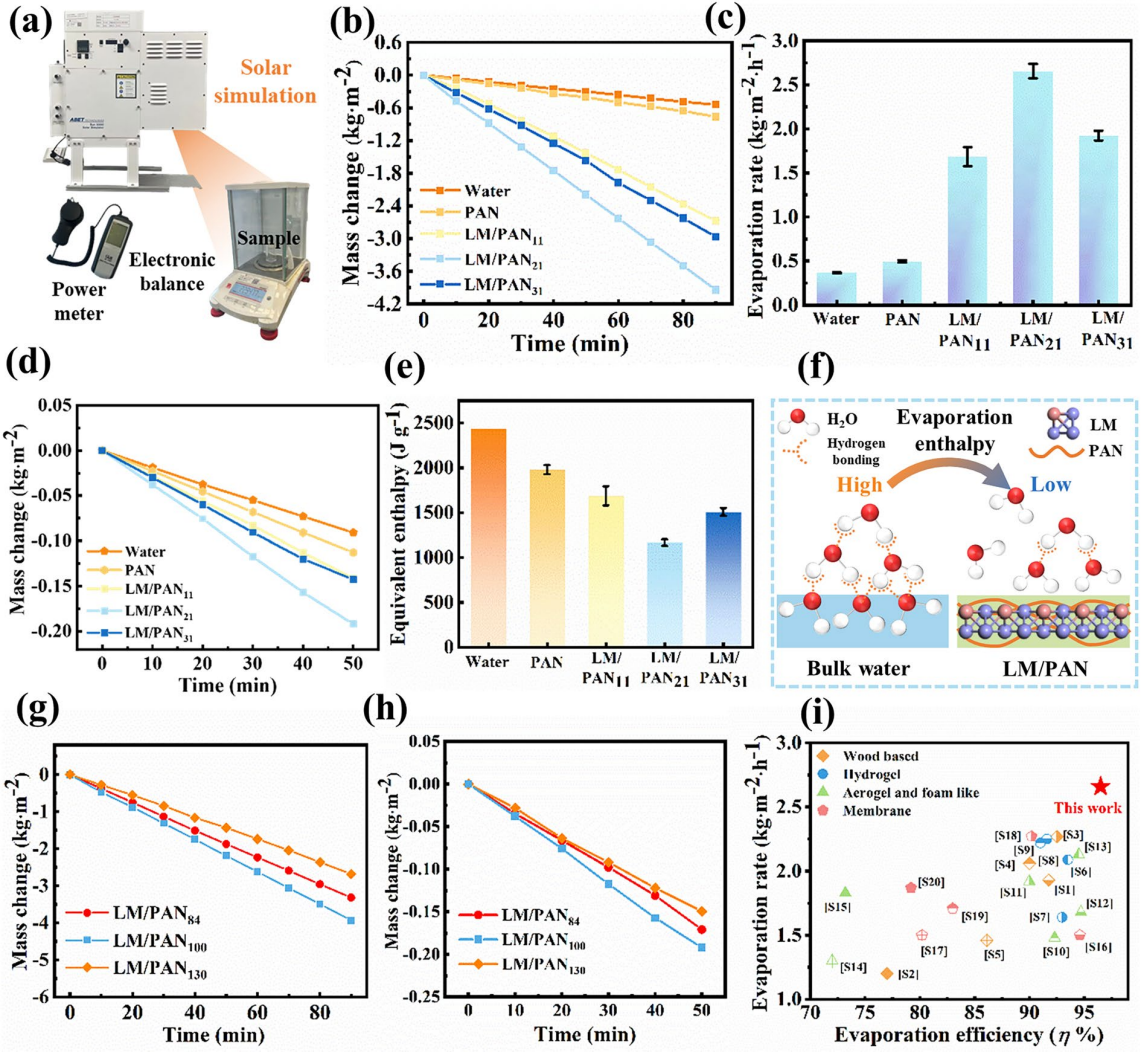
SSG performance of PAN evaporator and LM/PAN evaporators. **a** Schematic diagram of water evaporation experiments. **b** Change in mass of water **c** with corresponding evaporation rates of water, PAN evaporator and LM/PAN evaporators under one sun (1 kW m^−2^). **d** Mass loss of water, PAN evaporator and LM/PAN evaporators in dark condition experiment. **e** Equivalent evaporation enthalpy. **f** Schematic of reduced evaporation enthalpy in LM/PAN evaporators. Change in mass of LM/PAN_84_ evaporator, LM/PAN_100_ evaporator and LM/PAN_130_ evaporator **g** under 1 sun and **h** in the dark condition experiment. **i** Comparison with the performance of other kinds of SSG system under 1 sun

To evaluate the SSG performance of LM/PAN evaporators, the experimental apparatus of SSG was established as shown in Figs. 5a and S8. Under one sun radiation, the surface temperature of LM/PAN_31_ evaporator in the water exhibited a higher temperature (23.5 °C) than that of LM/PAN_21_ evaporator (21.2 °C), LM/PAN_11_ evaporator (18.4 °C) and PAN evaporator (5.3 °C) (Fig. S9). As shown in Fig. 5b, the water mass change of LM/PAN evaporators was higher than that of PAN evaporator and pure water. Moreover, LM/PAN_21_ evaporator showed a maximum water mass loss within 90 min compared to PAN evaporator and LM/PAN evaporators. The water evaporation rate was calculated and is plotted in Fig. 5c. LM/PAN_21_ evaporator presented a evaporation rate of 2.66 kg m^−2^ h^−1^, which was 1.39, 1.57, 5.91 and 6.33 times higher than that of LM/PAN_31_ evaporator (1.92 kg m^−2^ h^−1^), LM/PAN_11_ evaporator (1.69 kg m^−2^ h^−1^), PAN evaporator (0.50 kg m^−2^ h^−1^) and pure water (0.37 kg m^−2^ h^−1^), respectively. The notable SSG performance of LM/PAN_21_ evaporator was attributed to the synergistic effect of the efficient water transportation and excellent photothermal property. Although LM/PAN_31_ evaporator exhibited higher sunlight absorption compared with LM/PAN_21_ evaporator, the decreased porosity reduced the water transport ability, leading to lower SSG performance. Though PAN evaporator showed excellent water transportation capability with a saturated water content of 5.8 g g^−1^ in 20 s, poor sunlight absorption (5.1%) led to unsatisfactory SSG performance. In addition, the dark evaporation experiment (Fig. S10) conducted at 23 °C demonstrated that LM/PAN_21_ evaporator presented higher water evaporation performance than PAN evaporator and LM/PAN evaporators. According to the water mass change curve (Fig. 5d), the water evaporation rate (0.14 kg m^−2^ h^−1^) of PAN evaporator was close to that of pure water (0.10 kg m^−2^ h^−1^), while the water evaporation rate of LM/PAN evaporators significantly increased, indicating that the LM particles inside porous PAN greatly enhanced the SSG performance of LM/PAN evaporators. It can be seen that the LM/PAN_21_ evaporator displayed the higher mass change ratio of 0.23 kg m^−2^ h^−1^ than PAN evaporator and other kinds of LM/PAN evaporators. The phenomenon revealed that LM/PAN_21_ evaporator exhibited an optimized water content due to the hierarchical structure and the introduction of LM particles.

To further confirm the SSG performance, the water evaporation enthalpy and energy efficiency of LM/PAN evaporators were evaluated. Assuming that identical energy input (*U*_*in*_) needed for water evaporation under dark condition was constant, the equivalent water evaporation enthalpy (*∆H*_*eq*_) of PAN evaporators and LM/PAN evaporators was estimated as follows [47, 73]:

3 $$U_{in} = \Delta H_{we} m_{w} = \Delta H_{eq} m_{s}$$

where *ΔH*_*we*_ represents the evaporation enthalpy of pure water at 23 °C, *m*_*w*_ denotes the mass change ratio of bulk water and *m*_*s*_ is the mass change rate of PAN evaporator and LM/PAN evaporators. As shown in Fig. 5e, the evaporation enthalpy of water confined within PAN evaporator (1838.18 kJ kg^−1^) was smaller than that of bulk water** (**2439.50 kJ kg^−1^), indicating that the addition of LM particles further reduced the water evaporation enthalpy. Specifically, LM/PAN_21_ evaporator exhibited the lower value of 1166.72 kJ kg^−1^ compared with LM/PAN_11_ evaporator (1687.7 kJ kg^−1^) and LM/PAN_31_ evaporator (1507.56 kJ kg^−1^). To verify the decrease in the water evaporation enthalpy of PAN evaporator and LM/PAN evaporators, differential scanning calorimetric measurement (DSC) was taken (Fig. S11). It can be seen that a sharp heat flow peak at 65 − 75 °C followed by a rapid decrease in the bulk water, indicating the instantaneous water evaporation process. For PAN evaporator and LM/PAN evaporators, the heat flow signal showed a gradual decay after reaching the peak (Fig. S11a). The corresponding water evaporation enthalpy was calculated via the integration of heat flux over the time range (Table S1**)**. LM/PAN_21_ evaporator exhibited a lower value (1386.87 kJ kg^−1^) than LM/PAN_31_ evaporator, LM/PAN_11_ evaporator, PAN evaporator and pure water, which was consistent with the trend in evaporation under dark condition. The significantly reduced water evaporation enthalpy of LM/PAN_21_ evaporator accelerated the water evaporation process, thereby leading to the significant increase in the water evaporation rate. Based on the calculated *∆H*_*eq*_, the corresponding efficiency (*η*) for SSG was estimated according to Eqs. 1 and 2. All LM/PAN evaporators demonstrated higher solar evaporation efficiency than PAN evaporator (48.7%). Notably, the LM/PAN_21_ evaporator revealed solar evaporation efficiency of 96.5% in contrast to LM/PAN_11_ evaporator (85.1%) and LM/PAN_31_ evaporator (84.0%). The high evaporation efficiency was attributed to the synergistic effect of the excellent photothermal conversion of LM particles and the reduced heat loss due to the isolation between water transport channel and water evaporation area.

To explore the mechanism of the reduction in the water evaporation enthalpy, the water content inside PAN evaporator and LM/PAN_21_ evaporator was investigated. The IW/FW ratio during SSG in bulk water, PAN evaporator and LM/PAN_21_ evaporator was measured according to the Raman spectra. As shown in Fig. S12, the peaks of water molecules locate at 3200–3400 and 3500–3600 cm^−1^, corresponding to FW and IW, respectively. It can be seen that the IW/FW ratio in PAN evaporator was 0.33 compared with zero in bulk water. Significantly, after introduction of LM particles, the IW/FW ratio reached 1.02 in LM/PAN_21_ evaporator, revealing the great improvement of IW content due to the synergistic effect of LM nanoparticle and PAN. The higher content of IW can effectively activate water molecules and improve the water evaporation rate. To further confirm the increasing IW content and reduction in the water vaporization enthalpy during the SSG in LM/PAN_21_ evaporator, MD simulation was performed to simulate the water evaporation process. The simulation model and detailed simulation steps are illustrated in Fig. S13. After the molecular dynamic simulation under NVT (constant-volume) ensemble for 500 ps (Fig. S14a-c), 93 water molecules escaped from the surfaces of LM/PAN, which was higher than that of PAN (74) and pure water (40), respectively, indicating a more efficient water evaporation process for LM/PAN. Additionally, the evolution of hydrogen bonds in water layers during water evaporation was calculated (Fig. S14d-f). The difference of hydrogen bond number on the surface of LM/PAN was estimated to 285, which was higher than that of PAN (254) and bulk water (54). Meanwhile, the intramolecular energy of the water layer on surface of LM/PAN was the lowest with a value of 3127.69 kcal mol^−1^ in contrast to that on the surface of pure water (3648.39 kcal mol^−1^) and PAN (3317.84 kcal mol^−1^). The number of hydrogen bonds decreased along with intramolecular energy, further indicating that the amount of IW increased after the addition of PAN and LM. Therefore, LM/PAN evaporators demonstrated a reduced evaporation enthalpy compared with the bulk water as shown in Fig. 5f, thereby significantly accelerating the water evaporation process.

Apart from the content of LM, the SSG performance of LM/PAN evaporator was also affected by the diameter of LM/PAN fibers. LM/PAN evaporators fabricated by wet spinning needles with diameters of 0.84, 1.00 and 1.30 mm were prepared and defined as LM/PAN_84_ evaporator, LM/PAN_100_ evaporator and LM/PAN_130_ evaporator_,_ respectively. Meanwhile, the weight ratio of LM to PAN in all LM/PAN evaporators was maintain at 2:1 (Fig. S15a, b). LM/PAN_84_ fibers showed a similar cross-sectional structure as LM/PAN_100_ fibers with channels connected by smaller pores. As the diameter of LM/PAN fibers increased to 1.30 mm, a layered structure was observed in LM/PAN_130_ fibers with a ringlike structure wrapping the central cylinder. Meanwhile, similar sponge-like structure with micron pores on the channel surface similar as that in LM/PAN_100_ fibers were found in LM/PAN_84_ fibers and LM/PAN_130_ fibers (Fig. S15c-f). Interestingly, the specific surface area also varied with the change of the diameter of LM/PAN fibers. As shown in Fig. S16a, b, LM/PAN_130_ fibers showed a highest value of 12.51 m^2^ g^−1^ in comparison with LM/PAN_84_ fibers (7.95 m^2^ g^−1^) and LM/PAN_100_ fibers (11.34 m^2^ g^−1^). However, as the diameter of the cross-sectional area increased, the capillary force decreased according to Hagen–Poiseuille law [74], thereby decreasing water absorption and water transportation rate. Furthermore, the hydrophilicity of LM/PAN fibers was also affected by the diameter. As shown in Figs. 3c and S15g-h, LM/PAN_100_ fibers showed a stronger hydrophilicity than LM/PAN_84_ fibers and LM/PAN_100_ fibers. Water droplets quickly infiltrated across the surface within 0.25 s for LM/PAN_100_ fibers. In comparison, the infiltration time increased in LM/PAN_84_ fibers (within 4 s) and LM/PAN_130_ fibers (within 0.5 s). The SSG performance of LM/PAN_84_ evaporator, LM/PAN_100_ evaporator and LM/PAN_130_ evaporator under 1 sun irradiation was then investigated, as shown in Fig. 5g. The evaporation rates of LM/PAN_84_ evaporator, LM/PAN_100_ evaporator and LM/PAN_130_ evaporator were calculated as 2.21, 2.66 and 1.76 kg m^−2^ h^−1^, respectively. The outstanding SSG performance of LM/PAN_100_ evaporator results from the superior water transportation as shown in Fig. S16c. LM/PAN_100_ evaporator demonstrated superior water absorption rate before 20 s and eventually presented a higher saturated water content than that of LM/PAN_84_ evaporator (3.42 g g^−1^) and LM/PAN_130_ evaporator (3.33 g g^−1^). Besides, LM/PAN_100_ evaporator showed a higher evaporation rate of 0.23 kg m^−2^ h^−1^ under dark evaporation experiment than LM/PAN_84_ evaporator (0.20 kg m^−2^ h^−1^) and LM/PAN_130_ evaporator (0.18 kg m^−2^ h^−1^) (Fig. 5h). Therefore, relevant equivalent vaporization enthalpies were calculated as 1401.50, 1166.72 and 1489.47 kJ kg^−1^ for LM/PAN_84_ evaporator, LM/PAN_100_ evaporator and LM/PAN_130_ evaporator_,_ respectively. DSC measurement further confirmed the superior performance of LM/PAN_100_ evaporator on reducing the equivalent evaporation enthalpy (Fig. S16d and Table S2). Specifically, the LM/PAN_100_ exhibited a lower evaporation enthalpy of 1386.87 kJ kg^−1^ than that of LM/PAN_84_ (1584.03 kJ kg^−1^) and LM/PAN_130_ (1690.17 kJ kg^−1^). Consequently, the unique hierarchical porous structure of LM/PAN_100_ evaporator not only provided a favorable water transport channel, but also allowed the formation of large-size water clusters with low evaporation enthalpy, thereby significantly promoting the water evaporation.

Through tailoring the LM content and the diameter of LM/PAN fibers, the LM/PAN evaporator assembled by LM/PAN fibers with a diameter of 1 mm at a LM-to-PAN weight ratio of 1:2 exhibited a significantly enhanced SSG performance. Moreover, LM/PAN evaporator showed excellent evaporation ratio (2.66 kg m^−2^ h^−1^) and evaporation efficiency (96.5%), which are higher than previously reported wood-based, aerogel-based, membrane-based and hydrogel-based evaporator (Fig. 5i). Wood-based (green color) as well as aerogel-based evaporator (orange color) exhibited a comparable evaporation efficiency fluctuating in a wide range from 71% to 95%, but showed a moderate evaporation rate (~ 2.3 kg m^−2^ h^−1^). Membrane-based evaporator (pink color) presented a much lower evaporation rate (~ 1.9 kg m^−2^ h^−1^). Although hydrogel-based evaporator exhibited both high evaporation rate (~ 2.5 kg m^−2^ h^−1^) and efficiency (~ 93.5%), they suffered from complex fabrication processes as well as the unstable SSG performance.

### Practical SSG Application of LM/PAN Evaporators

**Fig. 6:**
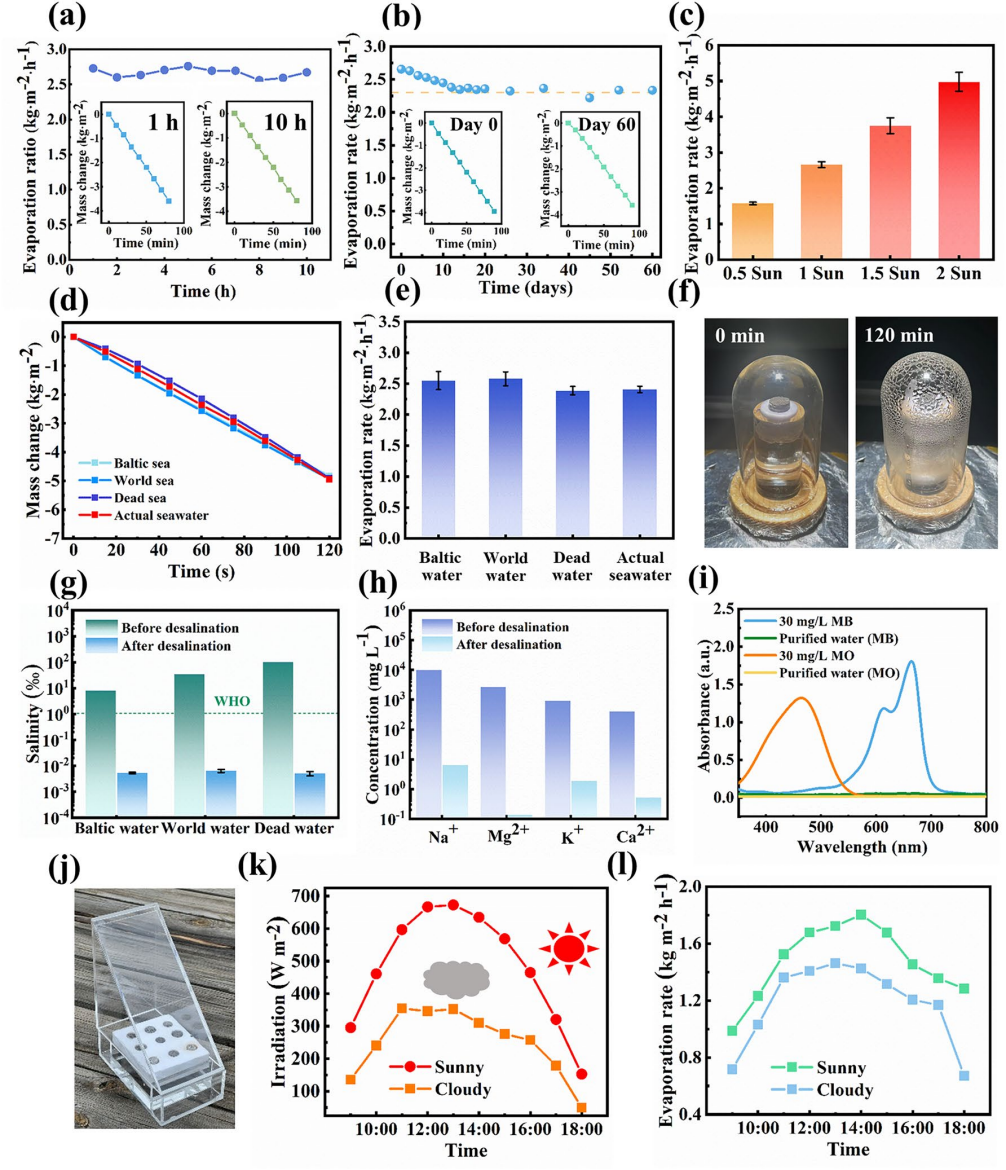
Practical SSG application of LM/PAN_21_ evaporator. **a** 10-h continuous evaporation test. **b** 60-day evaporation test of LM/PAN_21_ evaporator in water under 1 sun. **c** Water evaporation rates of LM/PAN_21_ evaporator under 0.5, 1, 1.5, 2 sun. **d** Mass change of three artificial seawater **e** with corresponding water evaporation rates of LM/PAN_21_ evaporator under 1 sun. **f** Optical images of the collection for condensation vapor. **g** Salinities of three artificial seawater before and after desalination using LM/PAN_21_ evaporator. The dash line is the World Health Organization (WHO) salinity standards for drinkable water. **h** Concentrations of Na^+^, Mg^2+^, K^+^ and Ca^2+^, in an actual seawater before and after desalination. **i** UV–Vis–NIR absorption spectra of MB and MO in water before and after evaporation using LM/PAN_21_ evaporator. **j** Outdoor SSG experiment. **k** Solar radiation from 09:00 to 18:00 on cloudy and sunny day and **l** the corresponding water evaporation rate

The stability of evaporators is crucial for practical applications. The LM/PAN_21_ evaporators were subjected to continuous testing under 1 sun for 10 h. As shown in Fig. 6a, the evaporation rate remained consistently around 2.65 kg m⁻^2^ h⁻^1^ throughout the testing period, with no significant performance degradation observed. After 10 h, the LM/PAN_21_ evaporator still demonstrated a remarkable evaporation rate of 2.67 kg m⁻^2^ h⁻^1^. The evaporation rate of LM/PAN_21_ evaporator demonstrated a slight decrease and then stabilized at approximately 2.34 kg m^−2^ h^−1^ under 1 sun for 60 days (Fig. 6b). Additionally, as shown in the Fig. S17, a freshly prepared LM/PAN_21_ evaporator with a density of 0.15 g cm^−3^ was able to support a 200 g weight without collapsing. Even after 10 h of continuous testing, the evaporator maintained its structural integrity, remaining capable of supporting the 200 g weight. Remarkably, even after 60 days, the evaporator retained its mechanical integrity and durability, as it could still withstand the 200 g weight following a 10-h evaporation test, confirming the exceptional long-term durability of the LM/PAN evaporators in terms of both solar steam generation and mechanical strength. Besides, the water evaporation rate of LM/PAN_21_ evaporator linearly increased with the sun intensity as shown in Fig. 6c. Even under 0.5 sun irradiation, LM/PAN_21_ evaporator exhibited an impressive water evaporation rate of 1.57 kg m^−2^ h^−1^. Notably, the evaporation rate exceeded 5.00 kg m^−2^ h^−1^ under 2 sun irradiations, indicating the capability of LM/PAN_21_ evaporator to perform effectively under varying solar intensities.

To demonstrate the solar desalination capability of the optimized LM/PAN_21_ evaporator in seawater, three brine solutions were prepared by dissolving NaCl into deionized water to simulate the real seawater of Baltic Sea (8‰ salinities), World Sea (35‰ salinities) and Dead Sea (100‰ salinities). Moreover, the SSG performance of LM/PAN_21_ evaporator in a real seawater from Dameisha Beach Park in China was evaluated as well. As shown in Fig. 6d, e, the water evaporation rate of LM/PAN_21_ evaporator was comparable for all the four kinds of seawater. It can be seen that the LM/PAN_21_ evaporator still exhibited an outstanding evaporation rate above 2.3 kg m^−2^ h^−1^ in all seawater. The evaporated water was collected using a homemade equipment as shown in Fig. 6g. The concentration of Na^+^ in artificial seawater was significantly reduced by three orders and was approximately two orders below the drinking water standard of World Health Organization (WHO) (1‰) [75]. Moreover, in real seawater, the concentration of Na^+^, Mg^2+^, K^+^ and Ca^2+^ dramatically decreased (~ 7 mg L^−1^) after desalination (Fig. 6h), which was smaller than the values achieved through membrane-based seawater desalination strategy (10–500 mg L^−1^) [47]. Meanwhile, concerning the issue of dye pollution in water, the dye removal ability of LM/PAN_21_ evaporator was investigated using methylene blue (MB) and methyl orange (MO) solution. MB is a representative cationic dye, while MO is an anionic dye. Interestingly, LM/PAN_21_ evaporator exhibited an adsorption behavior with dissolved organic dyes in water. As shown in Fig. 6i, the blue color and orange color of MB and MO faded away in the collected vapor during the evaporation, respectively. UV–Vis–NIR absorption spectra further confirmed the effective dye removal of LM/PAN_21_ evaporator, as the characteristic absorption peaks of MB (around 665 nm) and MO (around 464 nm) disappeared after purification. Moreover, the LM/PAN_21_ evaporator presented equivalent adsorption ability after washed for four times (Fig. S18a) and exhibited a similar evaporation rate of 2.64 kg m^−2^ h^−1^ in MB or MO solution after each cycle. The effective dye removal capability can be attributed to the polar nitrile (–CN) groups in PAN, which interacts with the positively charged organic dye such as MB and metal ions [76]. Furthermore, the porous structure cooperatively provides the extensive adsorption sites for dyes, enabling physical trapping of dye molecules like MO [77, 78].

The SSG performance of optimized LM/PAN_21_ evaporator under natural conditions was evaluated by outdoor experiments by a solar evaporation prototype (SEP). The optical image of SEP is shown in Fig. 6j. LM/PAN_21_ evaporators were floated in a container with pure water using a foam support. A beveled lid was assigned to the container, triggering the water vapor to slide down to the collected area. The outside SSG performance was evaluated on both cloudy and sunny days from 9 am to 6 pm (Fig. 6k). As shown in Fig. 6l, sun irradiation plays a crucial role in the SSG performance of SEP. Specifically, SEP exhibited a water evaporation rate of 1.18 kg m^−2^ h^−1^ on a cloudy day with an average sun irradiation of 250 W m^−2^, whereas the water evaporation rate reached 1.47 kg m^−2^ h^−1^ on a sunny day with an average sun irradiation of 484 W m^−2^. Although the water evaporation rate of SEP decreased compared to the indoor experiment due to reduced sun irradiation from solar absorption and reflection by the lid, excellent SSG performance was still obtained for practical application. To further evaluate the practicability of LM/PAN evaporator, cost-effectiveness defined as the evaporation rate dividing to the cost per square was introduced [79]. As shown in Table S4, the facile fabrication process and lightweight characteristic reduce the material consumption as well as the cost of LM/PAN evaporator, while maintaining the ideal evaporation ratio. Finally, LM/PAN evaporator exhibited a superior cost-effectiveness of 12.4. Furthermore, to assess the environmental impact of the LM/PAN_21_ evaporator, UV–Vis spectroscopy was performed on water that had been continuously soaked with the LM/PAN_21_ evaporator for 2 days, as shown in Fig. S19. The UV spectrum of the soaked water displayed no characteristic peaks corresponding to PAN (270 nm) or LM particles (450 nm), indicating that the LM/PAN_21_ evaporator operated without releasing detectable pollutants into the water. This finding highlights its potential as an environmentally sustainable option for solar steam generation systems. Life cycle assessment was further performed to analyze the practicality of the LM/PAN evaporator. As shown in Fig. S20, during the life cycle, the greenhouse gas, toxic materials as well as land source consumption maintained a very low level, revealing the sustainability of LM/PAN evaporator.

## Conclusion

In conclusion, inspired by the hierarchical water transport channels and effective thermal management in the stem of bird of paradise, a multiscale biomimetic evaporator composed of vertically aligned LM/PAN fibers with porous structure was fabricated. The LM/PAN evaporator exhibited an outstanding evaporation rate of 2.66 kg m^−2^ h^−1^ and solar energy efficiency of 96.5% under 1 sun radiation. Highly efficient SSG performance was attributed to effective energy confinement due to tailored water transportation in LM/PAN evaporator. Specifically, the hierarchical water pathways in LM/PAN evaporator regulate the water distribution, providing expedited water absorption and transportation. Moreover, MD simulation verified that the interaction between LM/PAN evaporator and bulk water contributed to tunable water content with significantly reduced vaporization enthalpy. Additionally, the synergistic effect of the effective photothermal conversion of LM particles and the porous structure in LM/PAN evaporator enabled an efficient solar energy utilization system for powering water evaporation. For practical applications, the unique hierarchical porous structure endows LM/PAN evaporator with excellent performance in seawater desalination as well as dye removal in wastewater. Meanwhile, LM/PAN evaporator exhibited an evaporation rate of 1.47 and 1.18 kg m^−2^ h^−1^ under an average solar illumination of 250 and 484 W m^−2^, respectively, in the outdoor test. The facile processing method, excellent SSG performance and desalination capability promote LM/PAN evaporator to be a potential candidate for addressing environmental problem, including environmental cooling, moisture management and pollution abatement.

## Supplementary Information

Below is the link to the electronic supplementary material.

Supplementary file1 (DOCX 12002 kb)
