# Supplementary material for: Multiscale Biomimetic Evaporators Based on Liquid Metal/Polyacrylonitrile Composite Fibers for Highly Efficient Solar Steam Generation
Source: Nanomicro Lett. 2025 Feb 5;17:129. doi: 10.1007/s40820-025-01661-z (PMC11799508; doi:10.1007/s40820-025-01661-z)
Supplement: Supplementary file 1 — Supplementary file1 (DOCX 12002 kb) [file 40820_2025_1661_MOESM1_ESM.docx]

# Supporting Information for

# Multiscale Biomimetic Evaporators Based on Liquid Metal/Polyacrylonitrile Composite Fibers for Highly Efficient Solar Steam Generation

Yuxuan Sun^1^, Dan Liu^1^, Fei Zhang^1^, Xiaobo Gao^1^, Jie Xue^1^, Qingbin Zheng^1,*^

^1^School of Science and Engineering, The Chinese University of Hong Kong, Shenzhen, Guangdong, 518172, P. R. China

*Corresponding author. E-mail: [zhengqingbin@cuhk.edu.cn](mailto:zhengqingbin@cuhk.edu.cn) (Qingbin Zheng)

**Supplementary Figures and Tables**


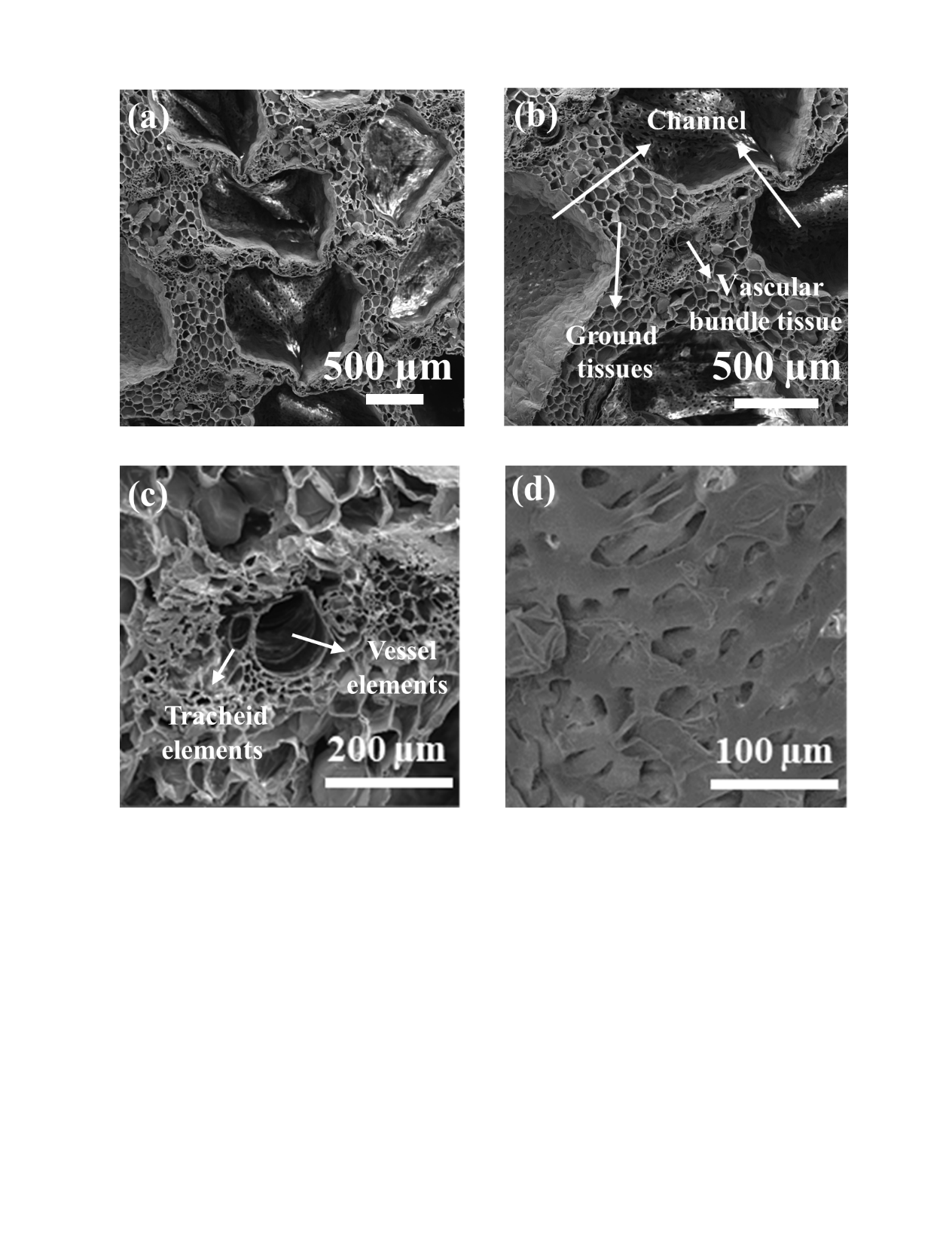


**Fig. S1** SEM images of **a** the cross-area of birds of paradise stem, **b** Ground tissues and vascular bundle tissue in the stem of birds of paradise. **c** Tracheid and vessel elements and **d** Porous structure at the channel surface

**
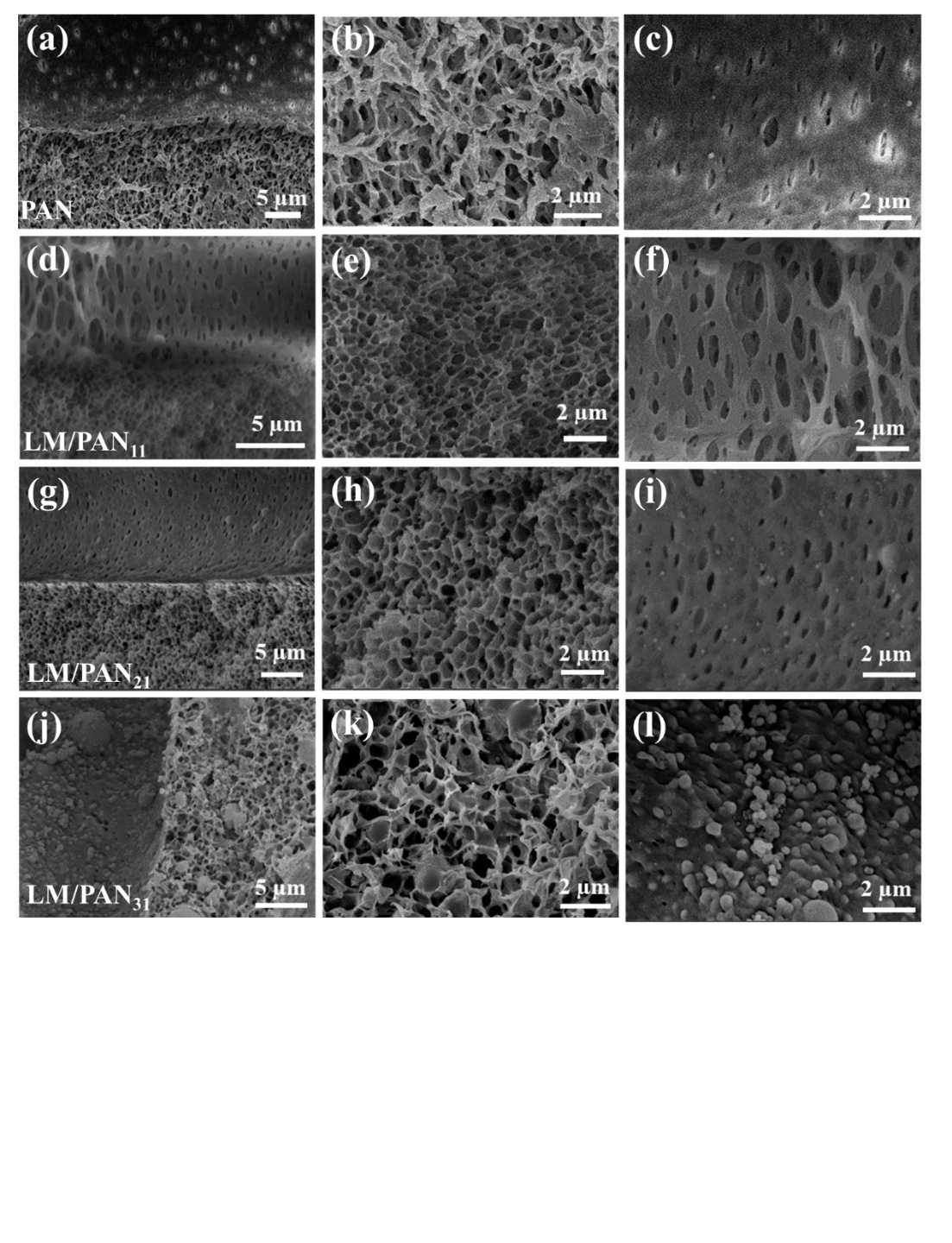
**

**Fig. S2** SEM images of a-**c** PAN, d-f) LM/PAN_11_. g-i) LM/PAN_21_ and j-l) LM/PAN_31_ fiber


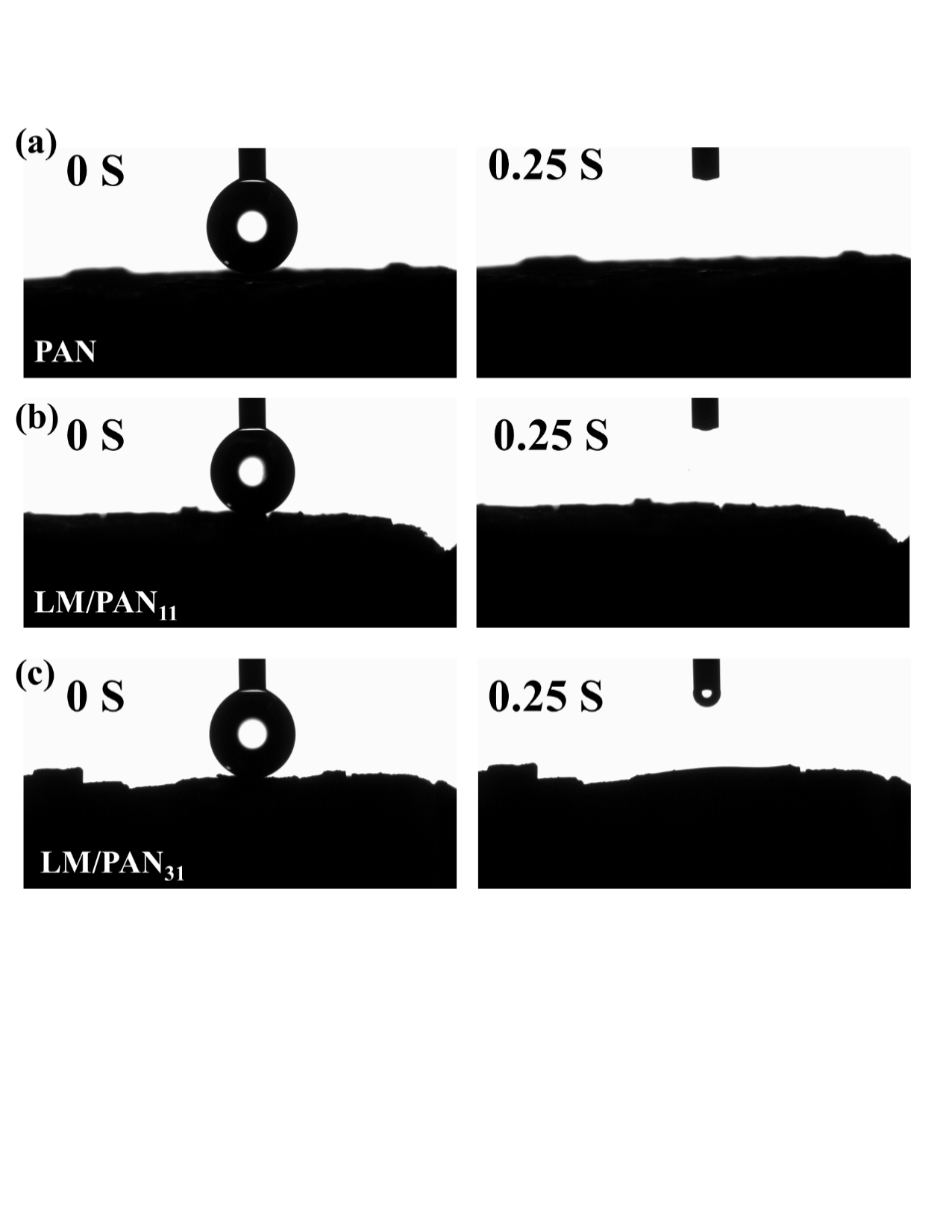


**Fig. S3** Water contact angle test of **a** PAN, **b** LM/PAN_11_ and **c** LM/PAN_31_ fibers


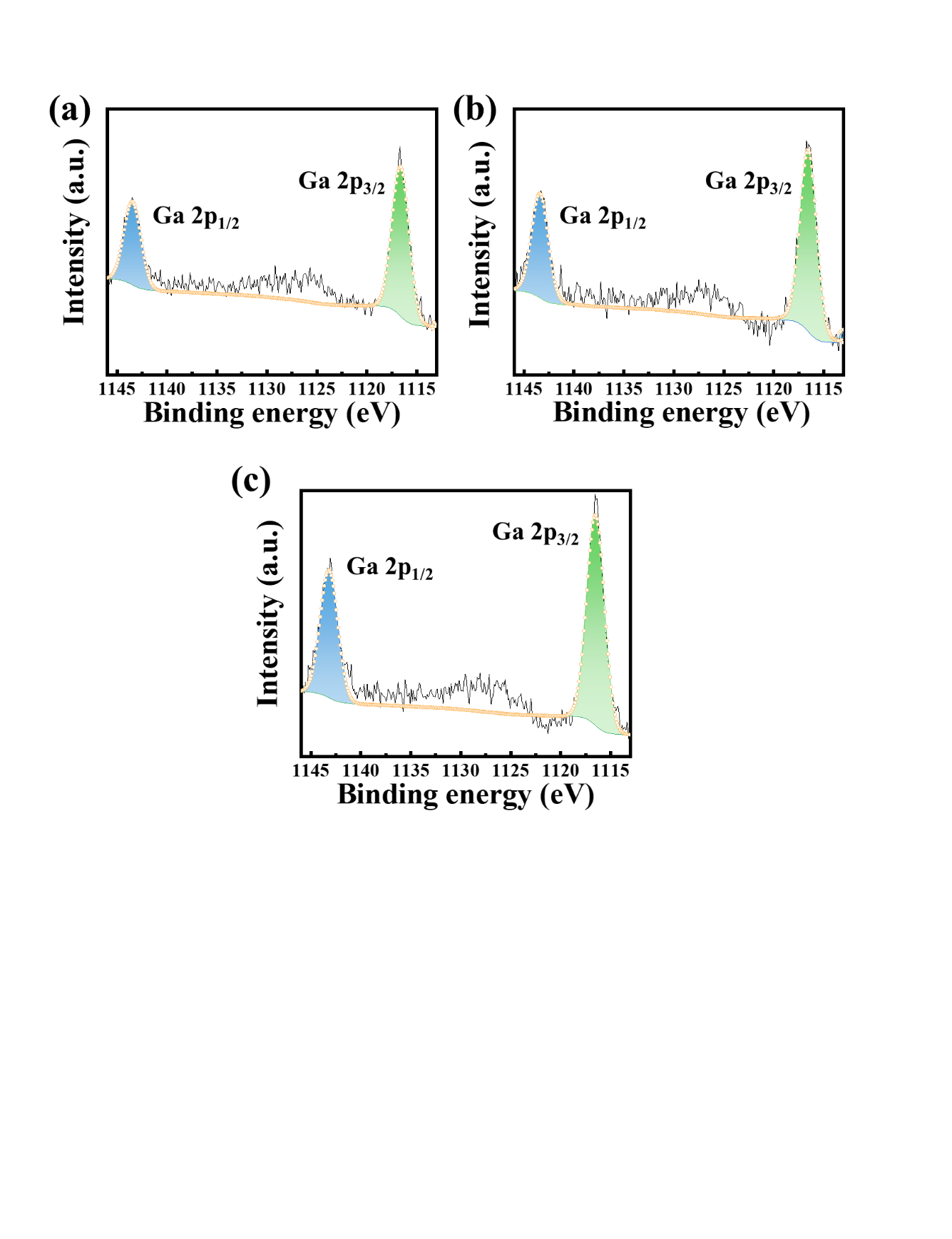


**Fig. S4** High-resolution XPS spectra of Ga 2p in LM/PAN_11_, LM/PAN_21_ and LM/PAN_31_ evaporators


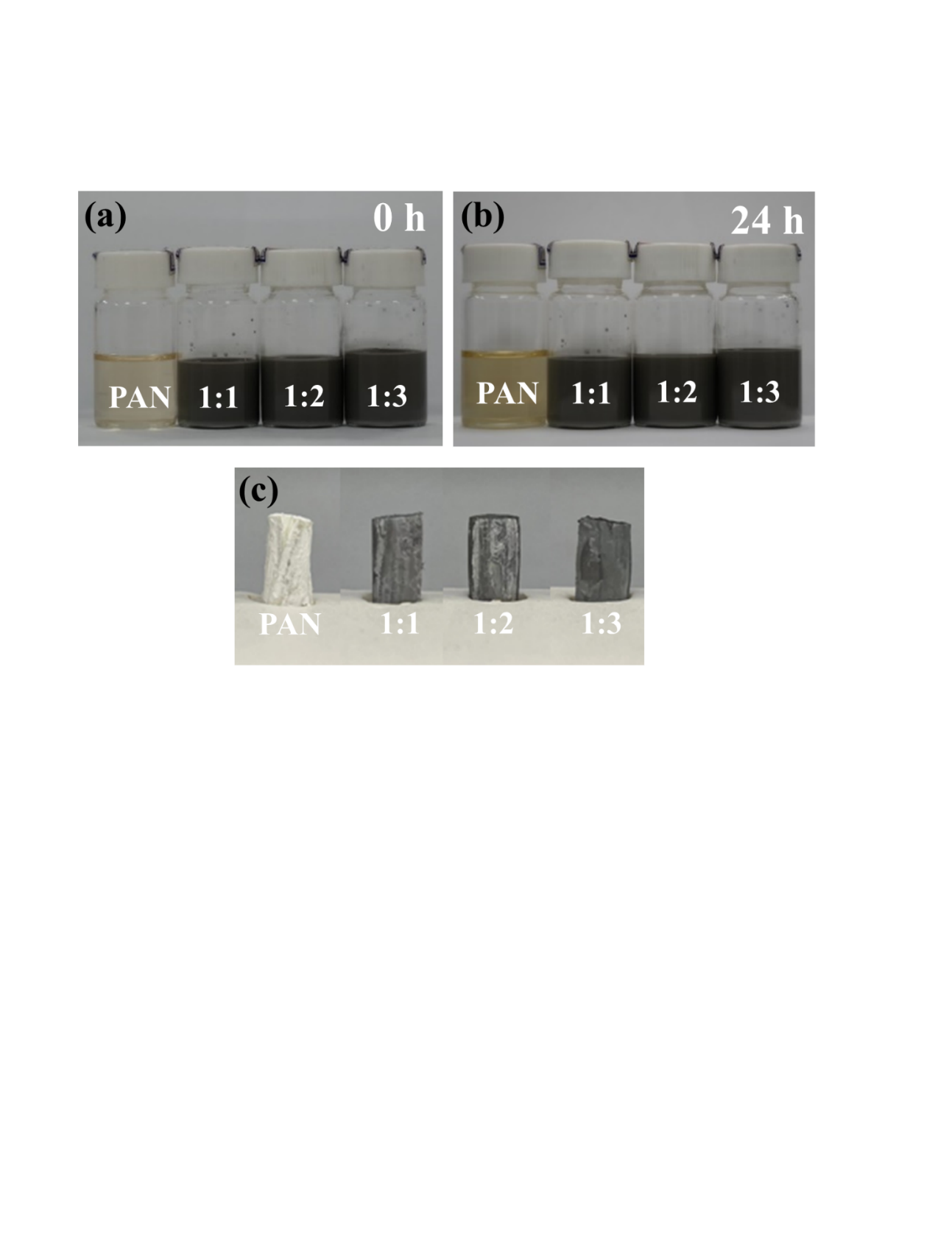


**Fig. S5** Optical images of **a** the wet spinning dope with a PAN to LM weigh ratio of 0, 1:1, 1:2 and 1:3, **b** the dope with a homogenous distribution after 24 h. **c** The prepared PAN, LM/PAN_11_, LM/PAN_21_ and LM/PAN_31_ evaporators


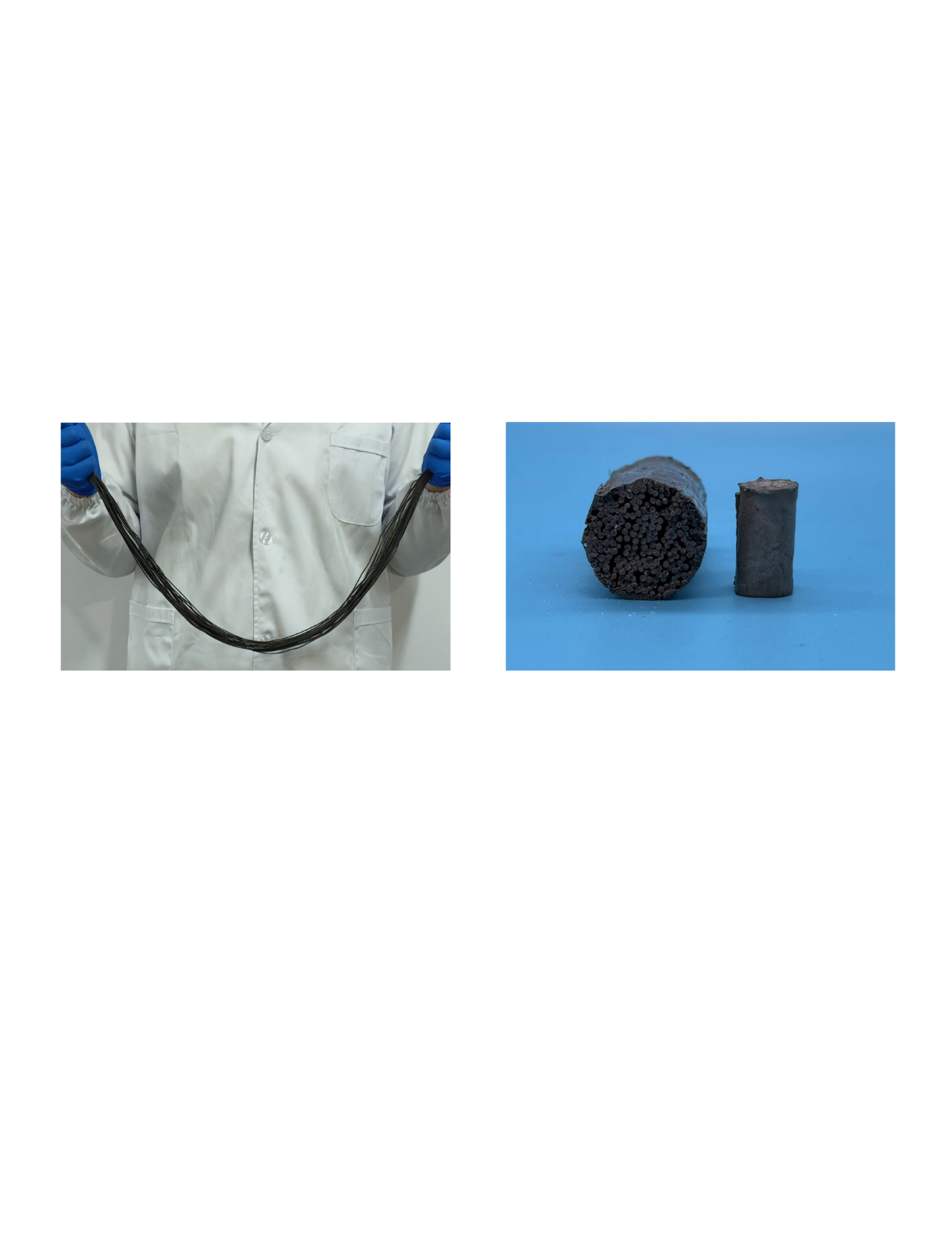


**Fig. S6** **a** Digital picture of the LM/PAN fiber. **b** LM/PAN evaporators with various configurations


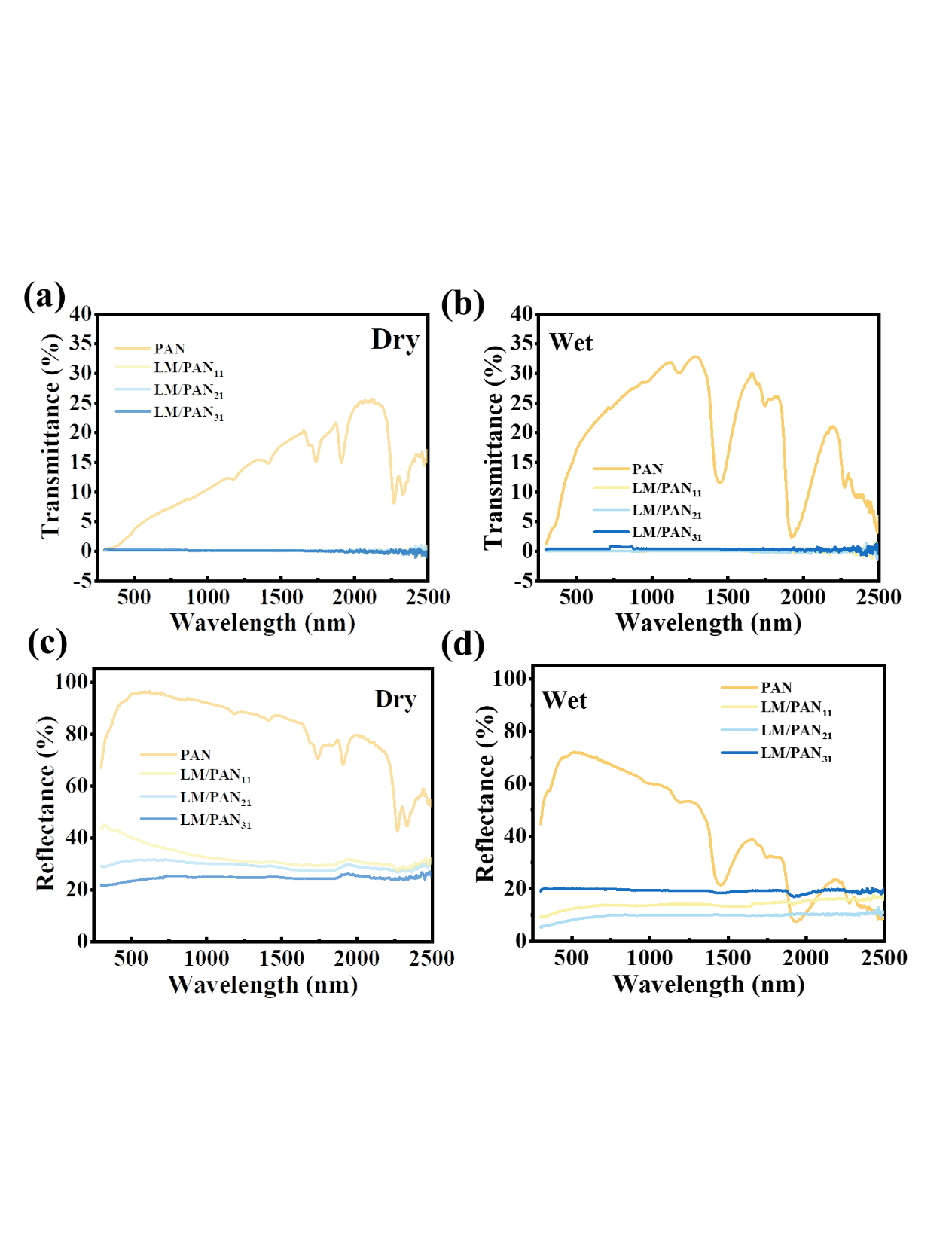


**Fig. S7** Reflectance spectra of (**a** dry and (**b** PAN, LM/PAN_11_, LM/PAN_21_ and LM/PAN_31_ evaporators. Transmittance spectra of **c** dry and **d** PAN, LM/PAN_11_, LM/PAN_21_ and LM/PAN_31_ evaporators


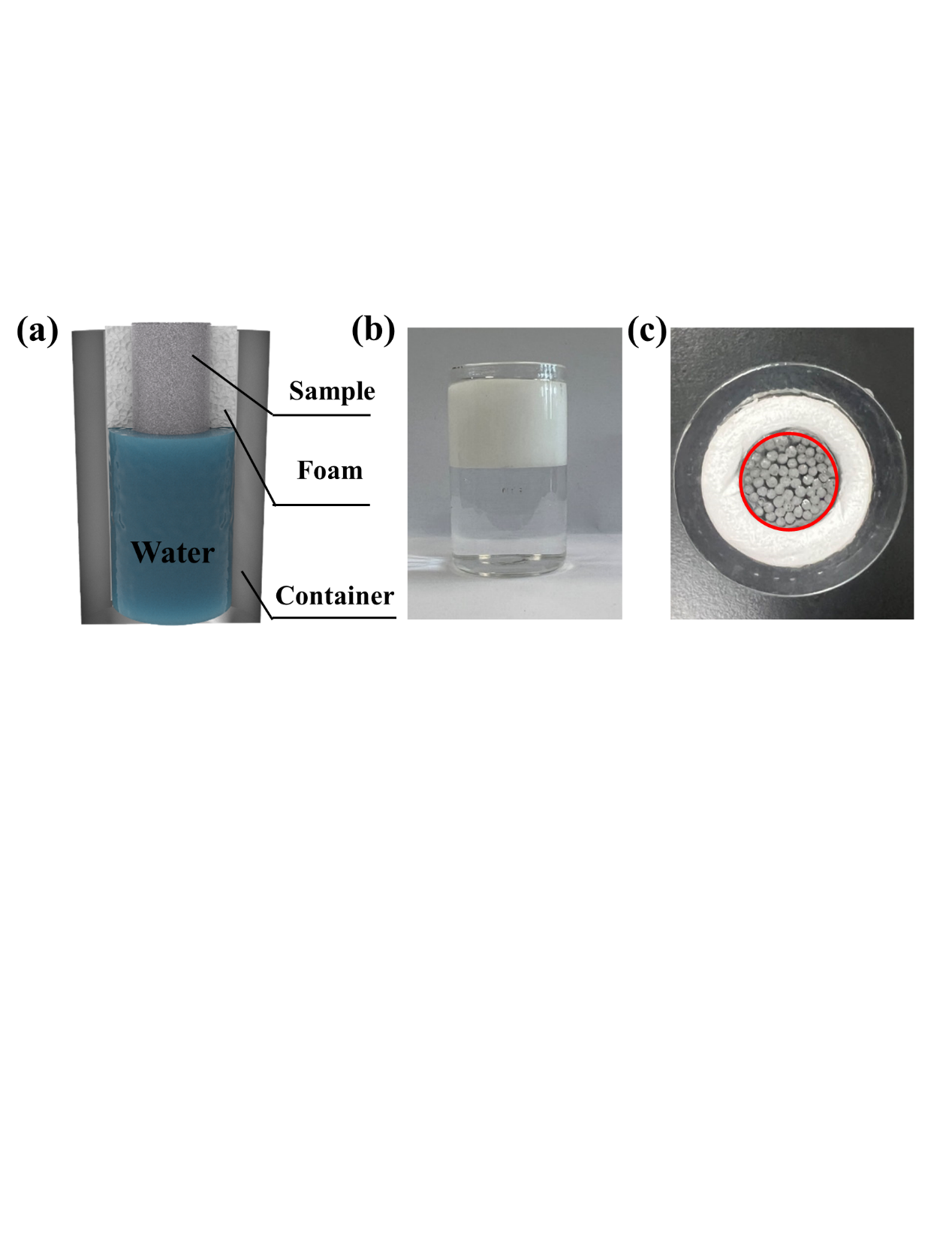


**Fig. S8** Schematic diagram of **a** apparatus for evaporation experiments and **b** relative optical image, **c** the surface area (red circle enclosed area of PAN and LM/PAN evaporators that used for calculate the evaporation rate)


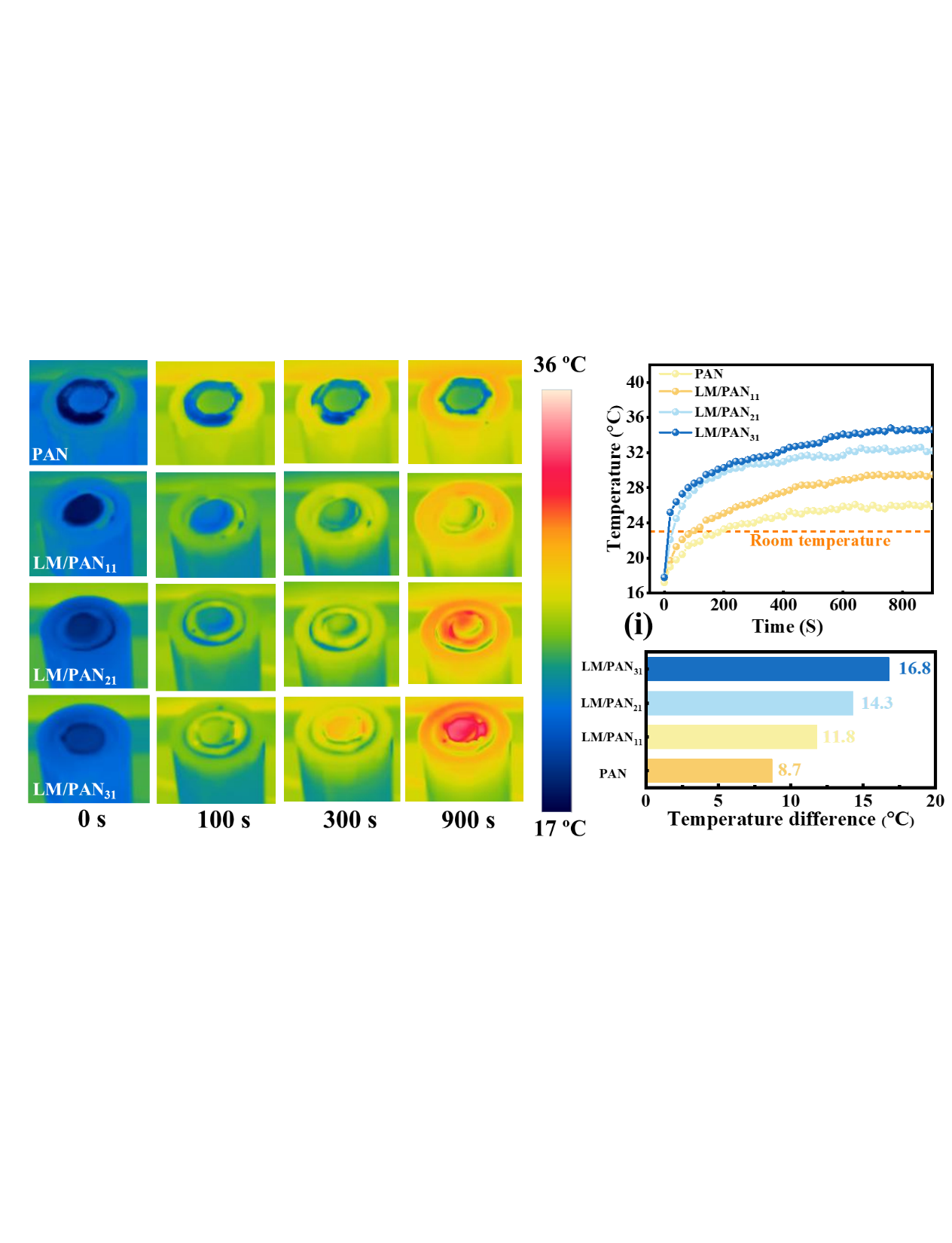


**Fig. S9** **a** IR image. **b** Top surface temperature evolution over time and **C** temperature difference between 0 s and 600 s in PAN and LM/PAN evaporators during the SSG experiment

**Estimation of Water Equivalent Evaporation Enthalpy**

*Dark evaporation experiment*: The dark evaporation experiment was performed according to a previous reported method [S1, S2]. PAN and LM/PAN evaporators were fixed in a polystyrene foam and placed in a bottle with enough supersaturated potassium carbonate solution as for experiments (**Fig. S10**). A sample of pure water with the comparable area was also prepared for comparison. The experiment was performed at 23 ºC and ambient air pressure. The loss weight of water of the apparatus was recorded per 10 minutes. Furthermore, the *∆H*_we_ of bulk water at 23 ºC can be calculated as follows [S3]:

${\Delta H}_{we}=C_{1}+C_{2}T+C_{3}T^{1.5}+C_{4}T^{2.5}+C_{5}T^{3}$ (S1)

where C_1_=2500.304, C_2_=-2.2521025, C_3_= -0.021465847, C_3_ =3.1750136×10^-4^, C_4_= -2.8607959×10^-5^ are constants, and *T* is temperature (°**C**. Thus, *∆H*_we_ of bulk water was calculated as 2439.5 kJ kg^-1^.


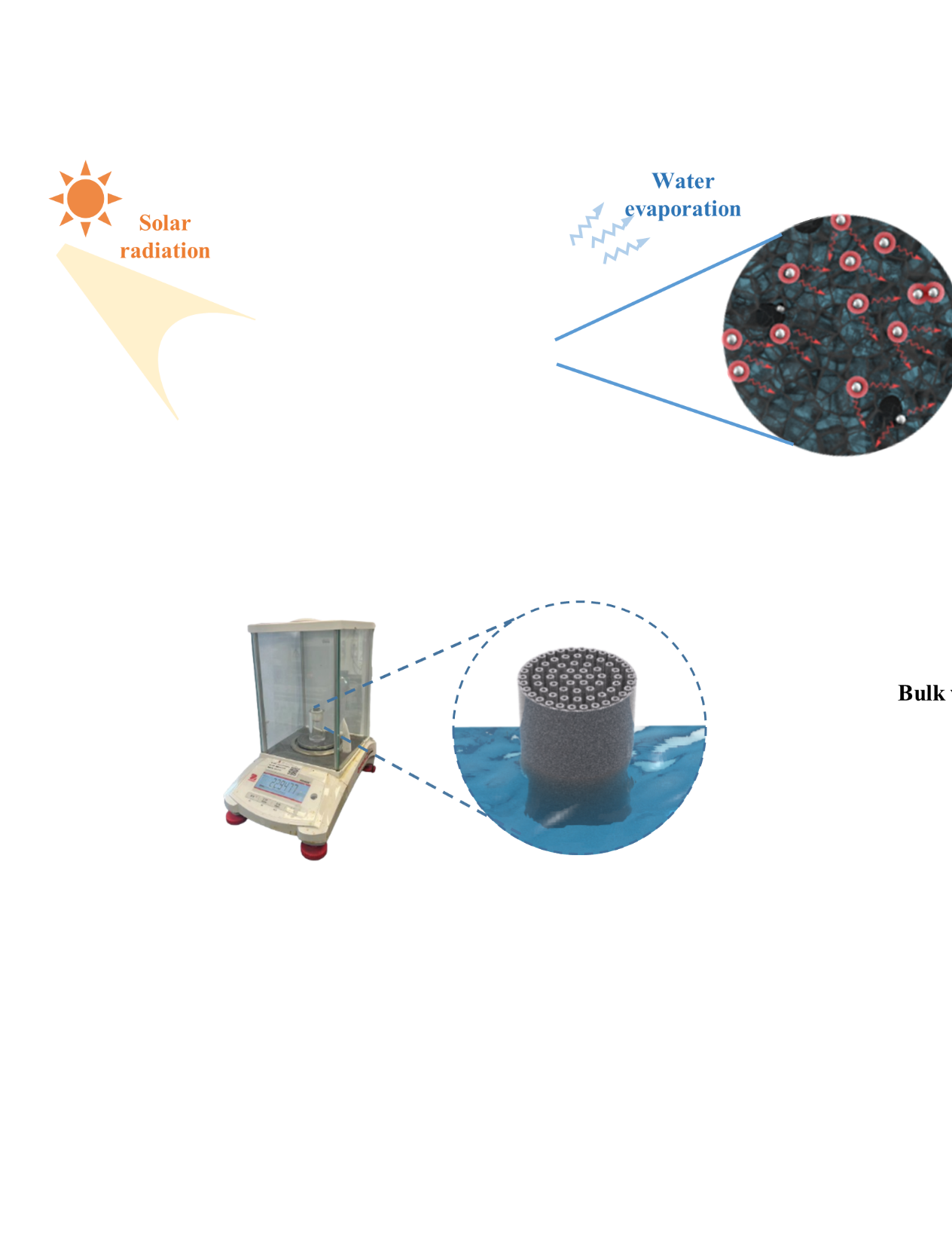


**Fig. S10** Schematic of dark evaporation experiment

*Water Equivalent Evaporation Enthalpy Measurement via DSC*: The original PAN and LM/PAN evaporators were cut and soaked into pure water for one day and the weight change was shown in **Table S1**. During water equivalent evaporation enthalpy measurement, the sample was placed in an open Al crucible and heated from 30 to 120 °C at a rate of 5 K/min under nitrogen atmosphere (30 mL/min). The water equivalent evaporation enthalpy was calculated based on the heat flow curves and shown in **Table S1**. It should be noted that the evaporation enthalpy in LM/PAN evaporators, calculated by DSC, was temperature-dependent and higher than that in the dark experiment. In the DSC tests, the selected temperature range was 30–120°C, whereas the actual temperature during practical applications was approximately 30°C. The difference in temperature contributes to the variation in the measured enthalpy values. Additionally, the DSC test represents complete dehydration from the swollen state, whereas the dark evaporation experiment involves a more gradual dehydration process. Therefore, the enthalpy value calculated through the dark evaporation experiment is more aligned with practical applications, where water molecules, with weaker hydrogen bonds, continuously diffuse toward the liquid-vapor interface from the water reservoir. Consequently, the water equivalent evaporation enthalpy obtained from DSC can only indicate the trend of enthalpy evolution across different samples, but cannot be used to calculate solar evaporation efficiency [S1, S3].

**
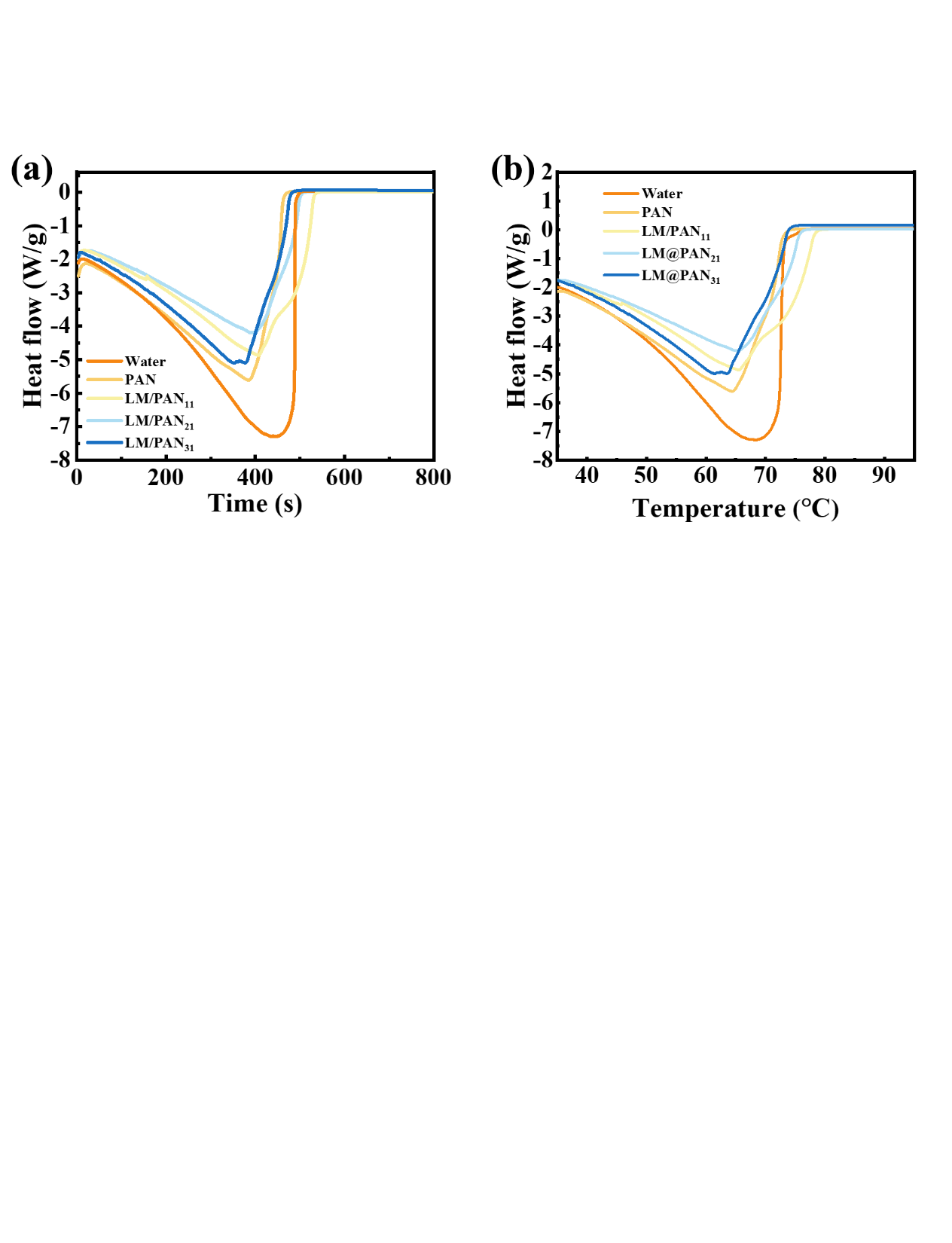
**

**Fig. S11** Heat flow signal as the function of **a** time and **b** temperature of water, PAN, LM/PAN_11_, LM/PAN_21_ and LM/PAN_31_ evaporator in the DSC measurement

**
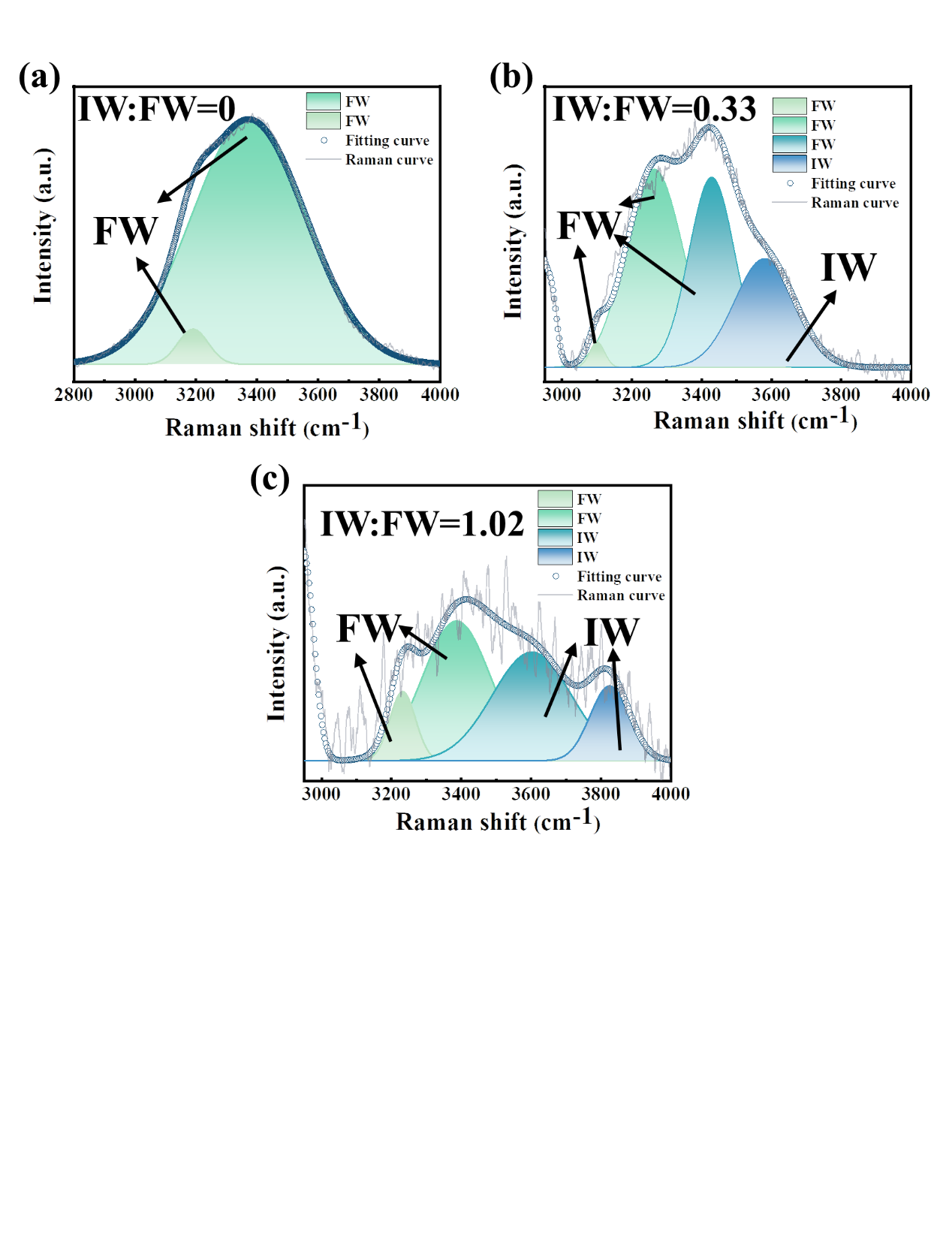
**

**Fig. S12** Fitting curves based on the Gaussian function in the energy region of O–H stretching modes of water in **a** bulk water, **b** PAN evaporator and **c** LM/PAN_21_ evaporator


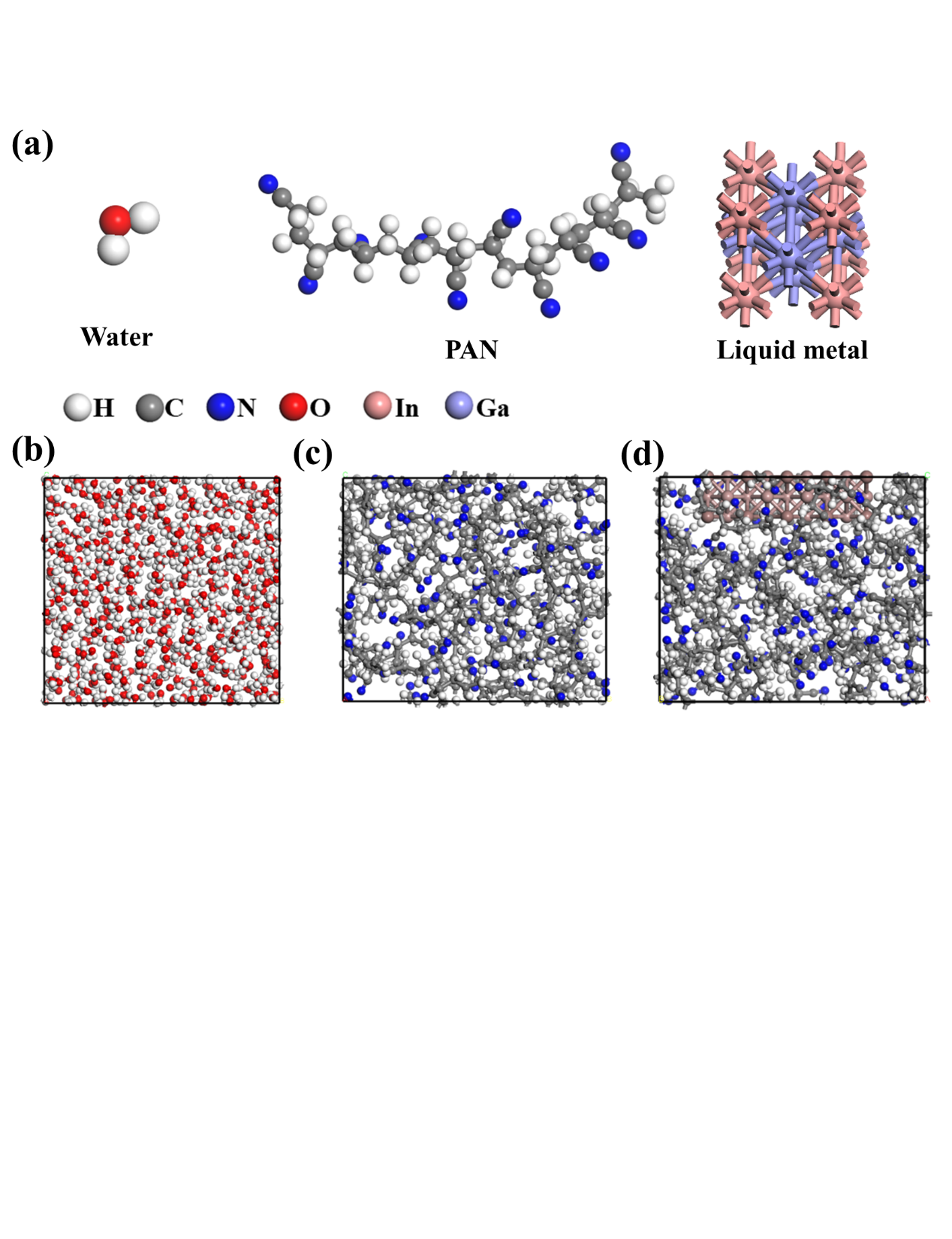


**Fig. S13** Simulation models of **a** water molecular, PAN chain, unit cell of LM, **c** water layer, PAN layer and LM/PAN layer used in dynamics simulation of the water evaporation process


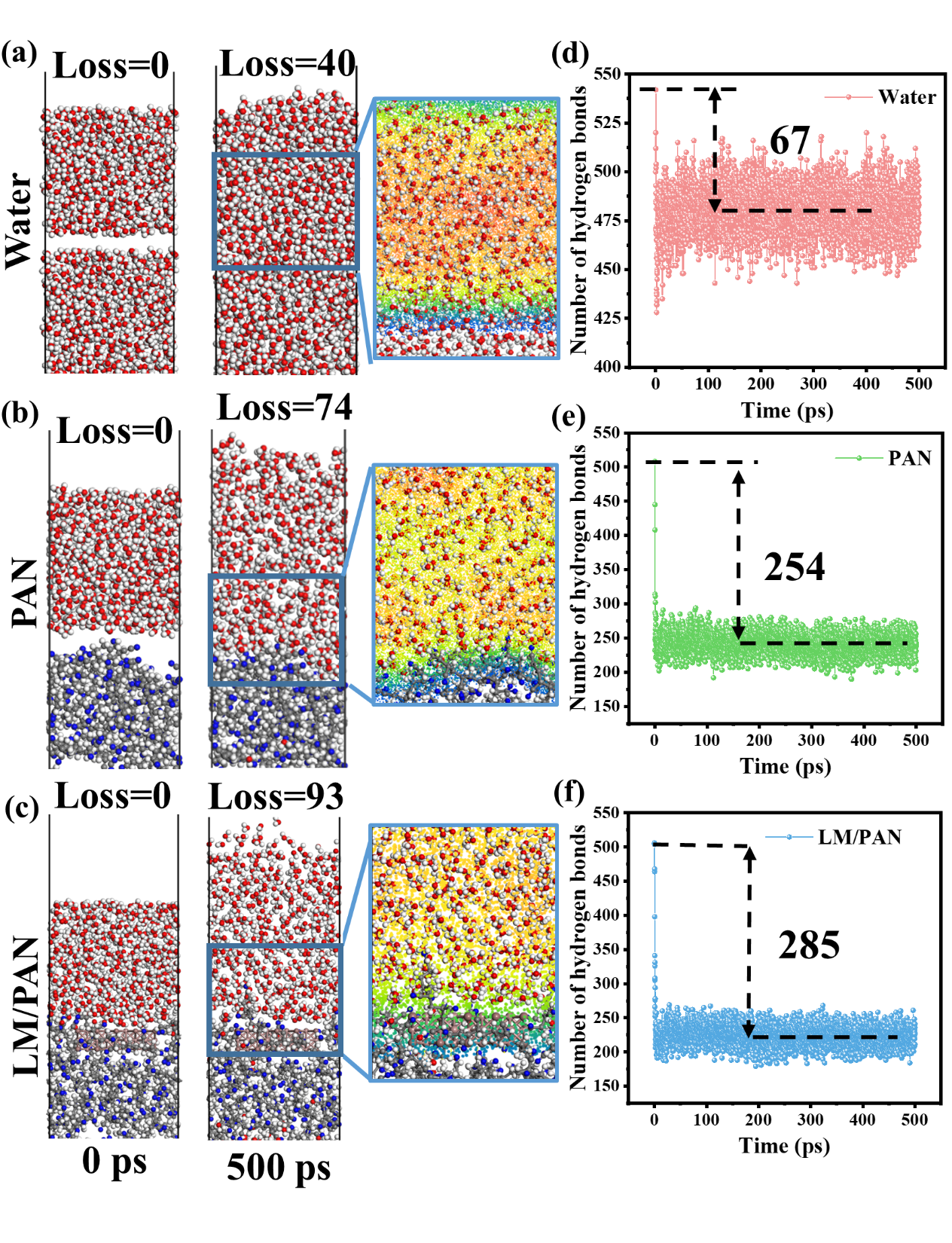


**Fig. S14** Water evaporation process of **a** pure water, **b** water in PAN, and **c** water in LM/PAN at 0, and 500 ps. The colourful insert images are the water molecular concentration at different evaporation interfaces. The hydrogen bonds number evolution in the 500 water molecules at the surface of **d** bulk water, **e** PAN, and f) LM/PAN during the evaporation process


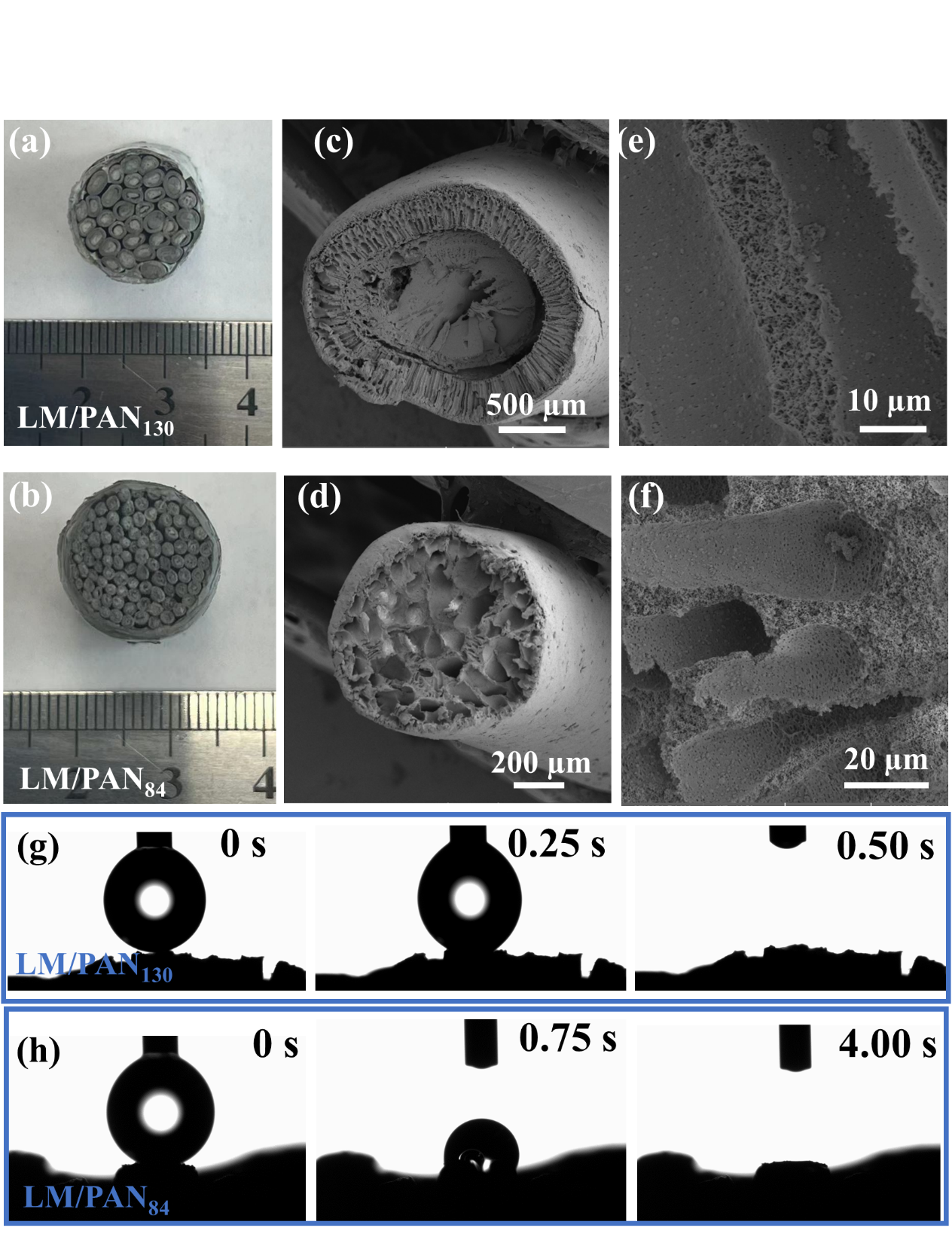


**Fig. S15** Optical image of **a** LM/PAN_84_ evaporator, **b** LM/PAN_130_ evaporator. SEM images of cross-area of fibers in **c** LM/PAN_84_ evaporator, **d** LM/PAN_130_ evaporator and relevant enlarged image **e** and **f**. Water absorption capability of **g** LM/PAN_84_ evaporator and **h** LM/PAN_130_ evaporator


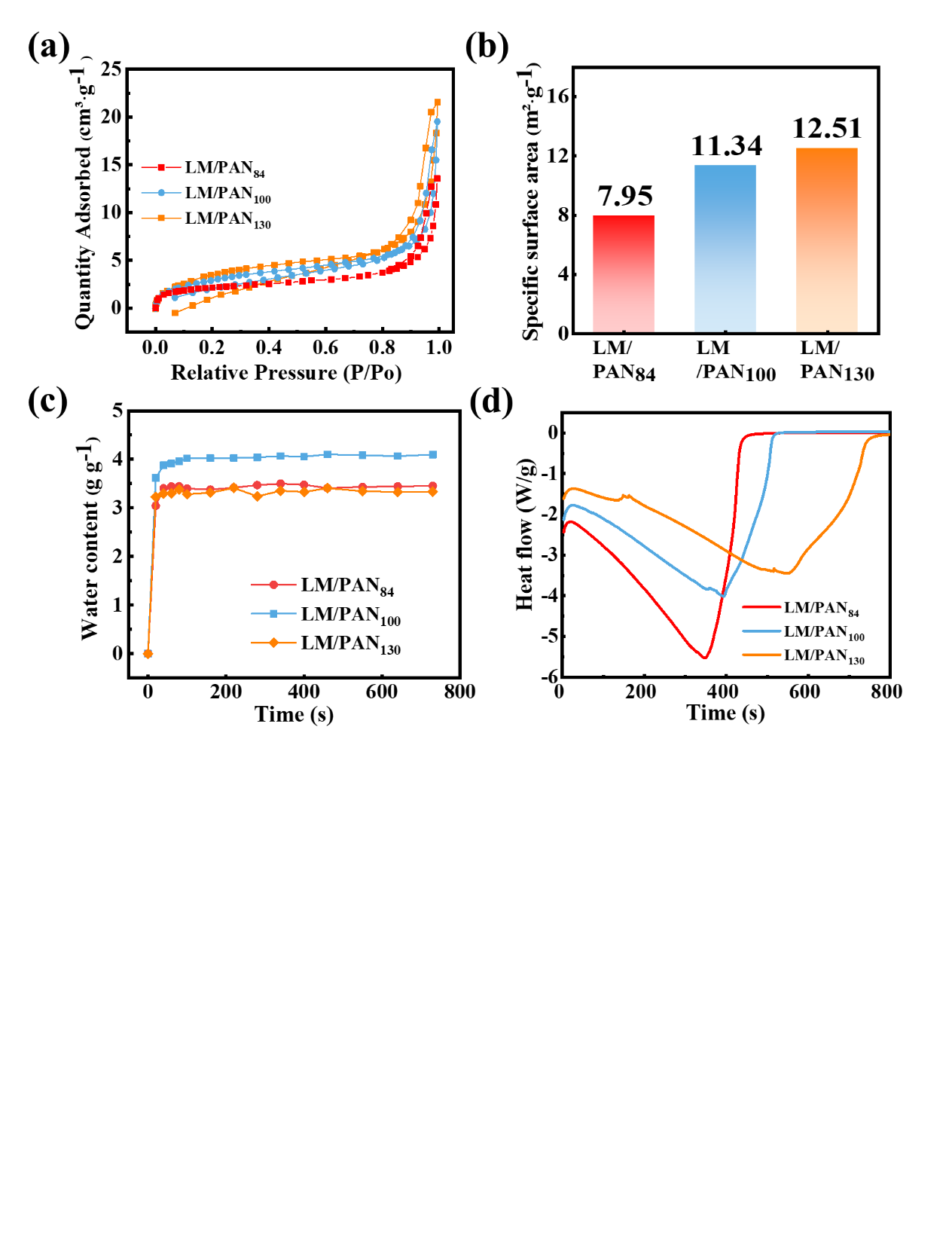


**Fig. S16 a** Nitrogen adsorption-desorption isotherms and **b** corresponding specific surface area of LM/PAN_84_, LM/PAN_100_ and LM/PAN_130_ evaporator. **c** Water content variation over time in LM/PAN_84_, LM/PAN_100_ and LM/PAN_130_ evaporator. **d** Heat flow signal as the function of time in LM/PAN_84_, LM/PAN_100_ and LM/PAN_130_ evaporator


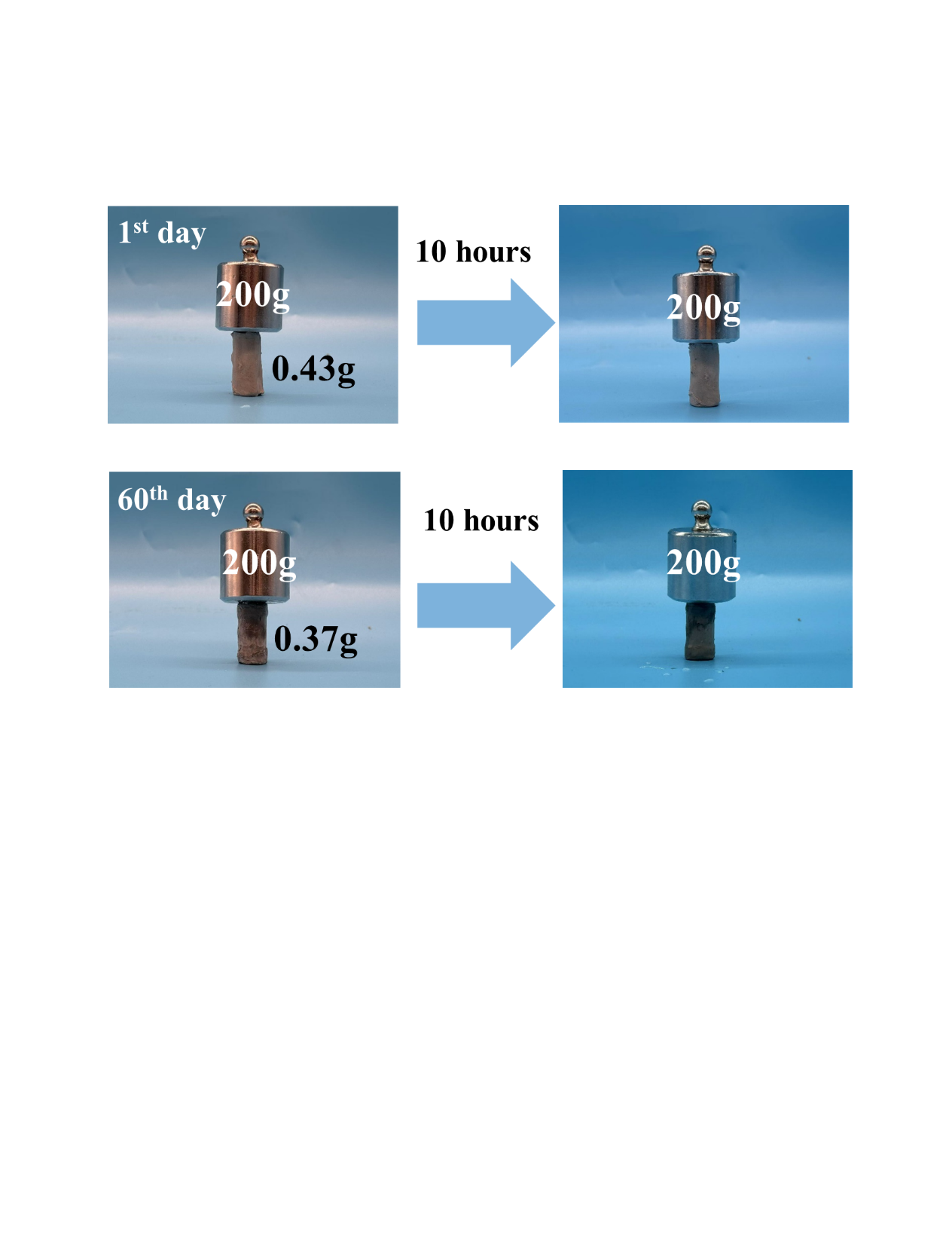


**Fig. S17** Mechanical strength of LM/PAN_21_ evaporator in 1st day and 60th day before and after 10 hours continuous evaporation test


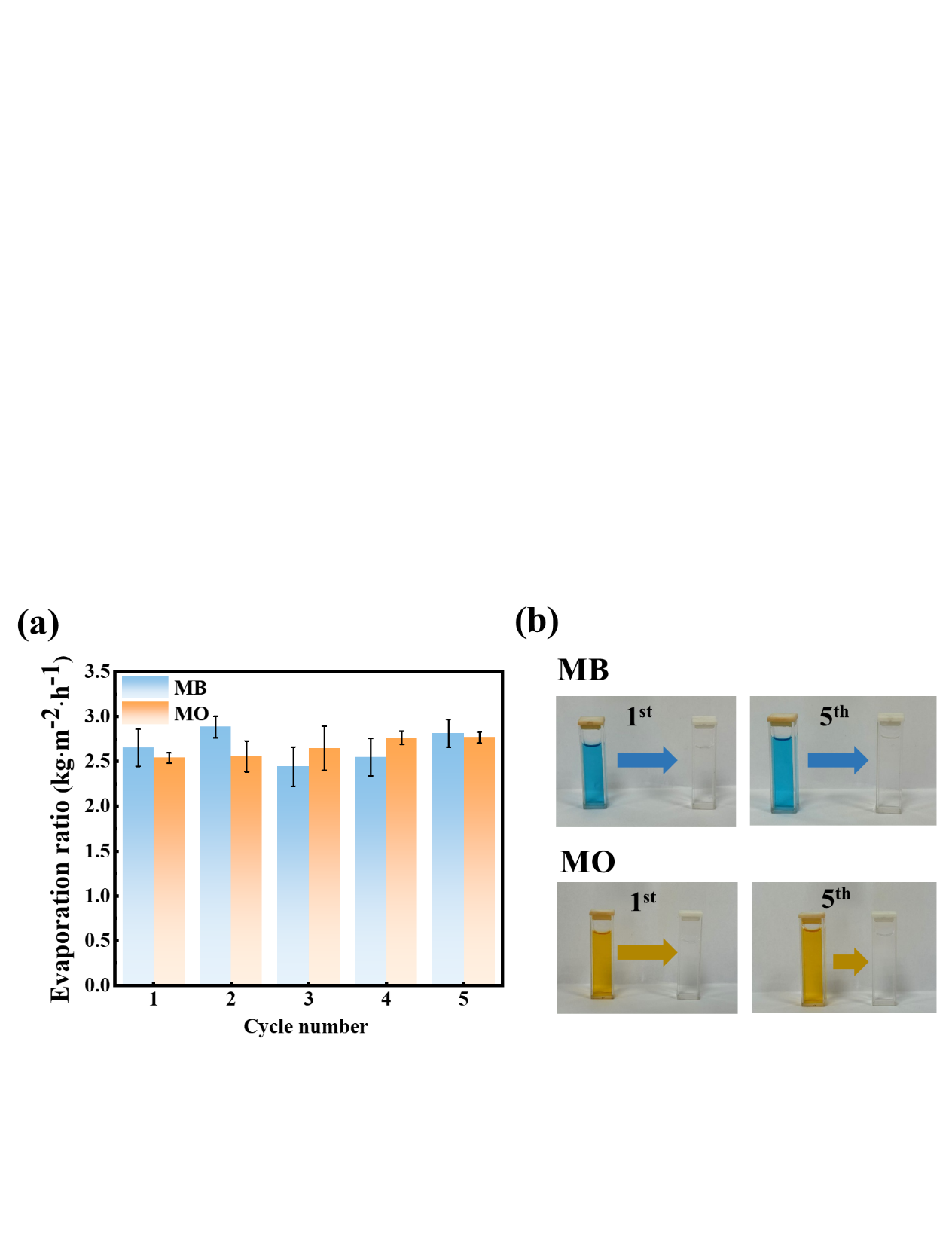


**Fig. S18 a** Water evaporation rates of LM/PAN_21_ evaporator in MB and MO solution after each cycle. **b** Optical images of dye solution and collected water in the 1st cycle and 5th cycle


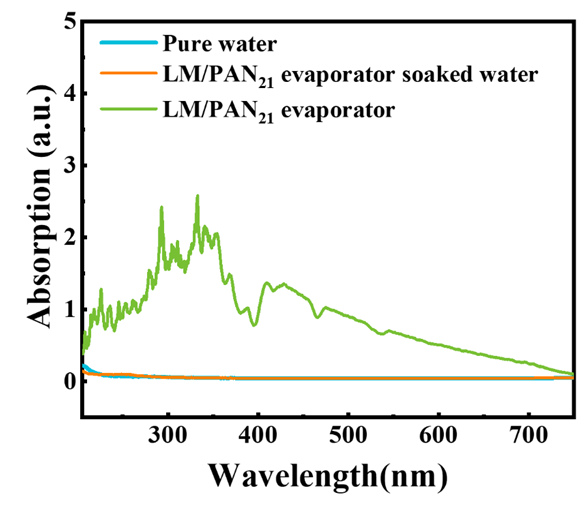


**Fig. S19** UV spectrum of pure water, LM/PAN_21_ evaporator, water that had been continuously soaked with the LM/PAN_21_ evaporator for 2 days

**Estimation the cost-effectiveness of LM/PAN evaporator**

LM/PAN evaporator was designed and prepared with a low density of 0.11 g cm^-3^, which ensures lower consumption of materials in practical production. Additionally, most of the raw materials, including PAN and DMF, are inexpensive and widely sourced. Cost-effectiveness was defined as the evaporation rate divided by the cost per unit area [S4]. Taking the LM/PAN_21_ evaporator as an example, the specific calculation is provided. To prepare a large-scale LM/PAN_21_ evaporator with a dimensional of (1 m^2^ 🞨2.5 cm), the total mass is 2.75 kg. The price of PAN powder is approximately 61.8 $ kg^-1^ (MACKLIN Co. Lt**d**, resulting in a cost of 56.7 $. The cost of DMF as the solvent is 7.4 $ for 8.25 kg (0.9 $ kg^-1^ for industrial grade DMF). The price of LM is 82.4 $ kg^-1^ (Dongguan Hua Titanium Material Technology Co. Lt**d**, so the cost is about 151 $ while consuming 1.83 kg. Taking all materials into account, the total cost is approximately 215.1$. Considering the evaporation rate of 2.66 kg m^-2^ h^-1^, the cost-effectiveness index is 12.4. The following **Table S4** presents a specific comparison of cost-effectiveness and solar vapor generation performance. Compared to other evaporator, LM/PAN evaporator exhibits superior photothermal evaporation performance while also offering favorable cost-effectiveness.

**Life cycle assessment**

To evaluate the life cycle assessment (LC**A** of LM/PAN evaporator, a preliminary LCA was performed using the open-source software OpenLCA, with data sourced from the ecoinvent database. For this evaluation, a LM/PAN evaporator with a demonsion of 1 m² × 2.5 cm was used. The assessment considered the raw materials, including liquid metal, PAN, DMF, and water, as well as the electricity consumed during the wet-spinning process. The emissions of greenhouse gases, the release of harmful substances, and the consumption of land and other resources throughout the evaporator's life cycle were quantified, and the results are presented in **Fig. S20**.


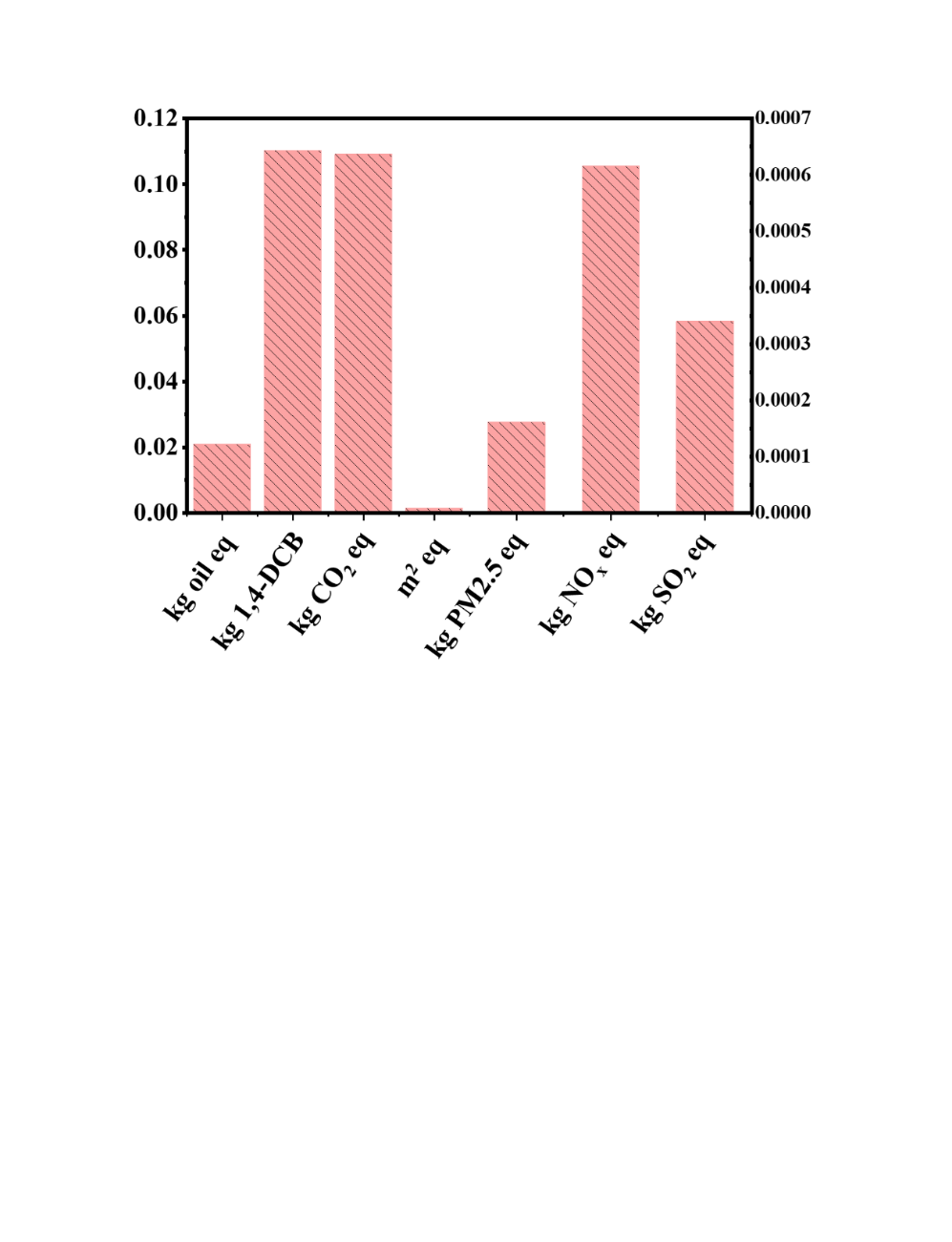


**Fig. S20** LCA of LM/PAN evaporator

**Table S1** Parameters for DSC measurement and calculated evaporation enthalpy of water in bulk water, PAN, LM/PAN_11_, LM/PAN_21_ and LM/PAN_31_ evaporator

|  | Dry mass (mg) | Wet mass (mg) | Evaporation enthalpy (kJ kg^−1^) |
| --- | --- | --- | --- |
| Water | - | 7.33 | 2215.88 |
| PAN | 1.1 | 8.52 | 1691.62 |
| LM/PAN_11_ | 1.7 | 8.24 | 1648.87 |
| LM/PAN_21_ | 1.9 | 8.25 | 1386.87 |
| LM/PAN_31_ | 1.6 | 6.80 | 1534.92 |

**Table S2** Parameters for DSC measurement and calculated evaporation enthalpy of water in LM/PAN_84_, LM/PAN_100_ and LM/PAN_130_ evaporator

|  | | Dry mass (mg) | Wet mass (mg) | | Evaporation enthalpy (kJ kg^−1^) | | |  |
| --- | --- | --- | --- | --- | --- | --- | --- | --- |
| LM/PAN_84_ | | 1.6 | 5.47 | | 1584.03 | | |  |
| LM/PAN_100_ | | 1.9 | 8.25 | | 1386.87 | | |  |
| LM/PAN_130_ | | 2.6 | 11.8 | | 1690.17 | | |  |
| **Table S3** Comparison of different SSG systems | | | | | | | | |
|  | Materials | | | Evaporation rate  (kg m^-2^ h^-1^) | | *η* (%) | Refs: | |
| Wood developed structure | Lignin-Wood | | | 1.93 | | 91.74 | [S5] | |
|  | Polydopamine/  Wood | | | 1.2 | | 77 | [S6] | |
|  | Carbon dot/Wood | | | 2.27 | | 92.5 | [S7] | |
|  | Carbon nanoparticle/Wood | | | 2.06 | | 90 | [S8] | |
|  | Nigrosin/Wood | | | 1.46 | | 86.1 | [S9] | |
| Hydrogel | Ti_3_C_2_TX/rGO/PVA hydrogel | | | 2.09 | | 93.5 | [S10] | |
|  | Acetylene black  sodium alginate polyacrylamide/ hydrogel | | | 1.64 | | 93 | [S11] | |
|  | Ti_3_C_2_TX/rGO/PVA hydrogel | | | 2.22 | | 91 | [S12] | |
|  | Polypyrrole (PPy)/  lignin hydrogel | | | 2.25 | | 91.6 | [S13] | |
| Aerogel and foam like structure | Ti_3_C_2_TX/carbon aerogels | | | 1.48 | | 92.3 | [S14] | |
|  | MoS2-sodium alginate hydrogel wrapped  Melamine foam | | | 1.92 | | 90 | [S15] | |
|  | PPy/PVA-F/Janus aerogel | | | 1.68 | | 94.7 | [S16] | |
|  | Polyacrylonitrile/carbon nanotubes | | | 2.13 | | 94.5 | [S17] | |
|  | Carbon black nanoparticles/ polymethylmethacrylate/ polyacrylonitril | | | 1.3 | | 72 | [S18] | |
|  | Coconut husk | | | 1.83 | | 73.2 | [S19] | |
|  | LM/PAN | | | 2.66 | | 96.5 | This work | |
| Membrane | AuNPs/  carbonized organosilica microspheres | | | 1.5 | | 94.6 | [S20] | |
|  | Cu-CAT-1 nanorod arrays/  gelatin composite membrane | | | 1.5 | | 80.2 | [S21] | |
|  | Polyacrylonitrile@copper sulfide | | | 2.27 | | 90.2 | [S3] | |
|  | Cotton paper | | | 1.71 | | 83 | [S22] | |
|  | Graphene oxide/carbon nanotubes | | | 1.87 | | 79.2 | [S23] | |

**Table S4** Comparison of cost-effectiveness of different SSG systems

| Materials | | | Evaporation rate (kg m^-2^ h^-1^) | cost-effectiveness （g h^-1^ USD^-1^） | References |
| --- | --- | --- | --- | --- | --- |
| Cellulose-based fabric / expanded polystyrene | 0.6 | | 200 | [S24] |  |
| Titanium sesquioxide nanoparticles / poly(vinyl alcohol) | 3.6 | | 12.3 | [S25] |  |
| Gold film / High-purity aluminum foil | 0.67 | | 0.1 | [S26] |  |
| Cermet-coated copper sheet / polystyrene foam | 0.5 | | 9 | [S27] |  |
| Polypyrrole / Stainless steel | 0.92 | | 16.7 | [S28] |  |
| Polypyrrole / Airlaid paper | 1.35 | | 67.5 | [S29] |  |
| Mixed Metal Oxide / quartz glass fibrous filter membrane | 2.04 | | 4.6 | [S30] |  |
| AIE-active molecule / Olive oil / poly(vinylidene fluoride-co-hexafluoropropylen**e** | 1.52 | | 11.2 | [S31] |  |
| Attapulgite / Acrylamide/ N, N′-Methylene bisacrylamide / polyvinyl pyrrolidone / polyvinyl alcohol | 1.2 | | 32.69 | [S32] |  |
| SiO_2_ / m-TiO_2–x_ | 1.05 | | 32.81 | [S33] |  |
| Polypyrrole / Ag | 1.55 | | 29.25 | [S34] |  |
| **LM / PAN** | 2.66 | | 12.4 | Our work |  |

**Supplementary References**

[S1] F. Zhao, X. Zhou, Y. Shi, X. Qian, M. Alexander et al., Highly efficient solar vapour generation via hierarchically nanostructured gels. Nat. Nanotechnol. 13, 489–495 (2018). <https://doi.org/10.1038/s41565-018-0097-z>

[S2] X. Zhou, F. Zhao, Y. Guo, B. Rosenberger, G. Yu, Architecting highly hydratable polymer networks to tune the water state for solar water purification. Sci. Adv. **5**, eaaw5484 (2019). <https://doi.org/10.1126/sciadv.aaw5484>

[S3] Z. Liu, Z. Zhou, N. Wu, R. Zhang, B. Zhu et al., Hierarchical photothermal fabrics with low evaporation enthalpy as heliotropic evaporators for efficient, continuous, salt-free desalination. ACS Nano **15**, 13007–13018 (2021). <https://doi.org/10.1021/acsnano.1c01900>

[S4] Y. Guo, H. Lu, F. Zhao, X. Zhou, W. Shi et al., Biomass-derived hybrid hydrogel evaporators for cost-effective solar water purification. Adv. Mater. **32**, e1907061 (2020). <https://doi.org/10.1002/adma.201907061>

[S5] Y. Gu, D. Wang, Y. Gao, Y. Yue, W. Yang et al., Solar-powered high-performance lignin-wood evaporator for solar steam generation. Adv. Funct. Mater. **33**, 2306947 (2023). <https://doi.org/10.1002/adfm.202306947>

[S6] Y. Zou, P. Yang, L. Yang, N. Li, G. Duan et al., Boosting solar steam generation by photothermal enhanced polydopamine/wood composites. Polymer **217**, 123464 (2021). <https://doi.org/10.1016/j.polymer.2021.123464>

[S7] Q. Hou, C. Xue, N. Li, H. Wang, Q. Chang et al., Self-assembly carbon dots for powerful solar water evaporation. Carbon **149**, 556–563 (2019). <https://doi.org/10.1016/j.carbon.2019.04.083>

[S8] Z. Luo, D. Yang, J. Liu, H.-Y. Zhao, T. Zhao et al., Nature-inspired solar-thermal gradient reduced graphene oxide aerogel-based bilayer phase change composites for self-adaptive personal thermal management. Adv. Funct. Mater. **33**, 2212032 (2023). <https://doi.org/10.1002/adfm.202212032>

[S9] L. Li, Y. Jia, K. Zeng, Z. He, J. Xue et al., A simple, mild, and low-cost method for preparation of wood-nigrosine in solar-driven interfacial evaporation system. Energy Technol. **11**, 2300531 (2023). <https://doi.org/10.1002/ente.202300531>

[S10] W. Li, X. Li, W. Chang, J. Wu, P. Liu et al., Vertically aligned reduced graphene oxide/Ti3C2Tx MXene hybrid hydrogel for highly efficient solar steam generation. Nano Res. **13**, 3048–3056 (2020). <https://doi.org/10.1007/s12274-020-2970-y>

[S11] J. He, Y. Fan, C. Xiao, F. Liu, H. Sun et al., Enhanced solar steam generation of hydrogel composite with aligned channel and shape memory behavior. Compos. Sci. Technol. **204**, 108633 (2021). <https://doi.org/10.1016/j.compscitech.2020.108633>

[S12] Y. Lu, D. Fan, Y. Wang, H. Xu, C. Lu et al., Surface patterning of two-dimensional nanostructure-embedded photothermal hydrogels for high-yield solar steam generation. ACS Nano **15**, 10366–10376 (2021). <https://doi.org/10.1021/acsnano.1c02578>

[S13] S. Jiang, Z. Zhang, T. Zhou, S. Duan, Z. Yang et al., Lignin hydrogel-based solar-driven evaporator for cost-effective and highly efficient water purification. Desalination **531**, 115706 (2022). <https://doi.org/10.1016/j.desal.2022.115706>

[S14] Z. Liu, F. Wu, T. Lv, Y. Qu, Z. Zhang et al., Ti3C2TX/carbon aerogels derived from winter melon for high-efficiency photothermal conversion. Desalination **573**, 117207 (2024). <https://doi.org/10.1016/j.desal.2023.117207>

[S15] J. Xiao, Y. Guo, W. Luo, D. Wang, S. Zhong et al., A scalable, cost-effective and salt-rejecting MoS_2_/SA@melamine foam for continuous solar steam generation. Nano Energy **87**, 106213(2021). <https://doi.org/10.1016/j.nanoen.2021.106213>

[S16] B. Wen, X. Zhang, Y. Yan, Y. Huang, S. Lin et al., Tailoring polypyrrole-based Janus aerogel for efficient and stable solar steam generation. Desalination **516**, 115228 (2021). <https://doi.org/10.1016/j.desal.2021.115228>

[S17] Y. Liu, H. Liu, J. Xiong, A. Li, R. Wang et al., Bioinspired design of electrospun nanofiber based aerogel for efficient and cost-effective solar vapor generation. Chem. Eng. J. **427**, 131539 (2022). <https://doi.org/10.1016/j.cej.2021.131539>

[S18] W. Xu, X. Hu, S. Zhuang, Y. Wang, X. Li et al., Flexible and salt resistant Janus absorbers by electrospinning for stable and efficient solar desalination. Adv. Energy Mater. **8**, 1702884 (2018). <https://doi.org/10.1002/aenm.201702884>

[S19] T.T. Pham, T.H. Nguyen, T.A.H. Nguyen, D.D. Pham, D.C. Nguyen et al., Durable, scalable and affordable iron (III) based coconut husk photothermal material for highly efficient solar steam generation. Desalination **518**, 115280 (2021). <https://doi.org/10.1016/j.desal.2021.115280>

[S20] R. Cui, J. Wei, C. Du, S. Sun, C. Zhou et al., Engineering trace AuNPs on monodispersed carbonized organosilica microspheres drives highly efficient and low-cost solar water purification. J. Mater. Chem. A **8**, 13311–13319 (2020). <https://doi.org/10.1039/D0TA03850D>

[S21] X. Ma, Z. Li, Z. Deng, D. Chen, X. Wang et al., Efficiently cogenerating drinkable water and electricity from seawater *via* flexible MOF nanorod arrays. J. Mater. Chem. A **9**, 9048–9055 (2021). <https://doi.org/10.1039/D0TA11870B>

[S22] Y.L. Wang, G.J. Li, K.C. Chan, Cost-effective and eco-friendly laser-processed cotton paper for high-performance solar evaporation. Sol. Energy Mater. Sol. Cells **218**, 110693 (2020). <https://doi.org/10.1016/j.solmat.2020.110693>

[S23] J. Han, Z. Dong, L. Hao, J. Gong, Q. Zhao, Poly(ionic liqui**d**-crosslinked graphene oxide/carbon nanotube membranes as efficient solar steam generators. Green Energy Environ. **8**, 151–162 (2023). <https://doi.org/10.1016/j.gee.2021.03.010>

[S24] G. Ni, S.H. Zandavi, S.M. Javid, S.V. Boriskina, T.A. Cooper et al., A salt-rejecting floating solar still for low-cost desalination. Energy Environ. Sci. **11**, 1510–1519(2018). <https://doi.org/10.1039/c8ee00220g>

[S25] Y. Guo, X. Zhou, F. Zhao, J. Bae, B. Rosenberger et al., Synergistic energy nanoconfinement and water activation in hydrogels for efficient solar water desalination. ACS Nano **13**, 7913–7919(2019). <https://doi.org/10.1021/acsnano.9b02301>

[S26] K. Bae, G. Kang, S.K. Cho, W. Park, K. Kim et al., Flexible thin-film black gold membranes with ultrabroadband plasmonic nanofocusing for efficient solar vapour generation. Nat. Commun. **6**, 10103(2015). <https://doi.org/10.1038/ncomms10103>

[S27] G. Ni, G. Li, S.V. Boriskina, H. Li, W. Yang et al., Steam generation under one Sun enabled by a floating structure with thermal concentration. Nat. Energy **1**, 16126(2016). <https://doi.org/10.1038/nenergy.2016.126>

[S28] L. Zhang, B. Tang, J. Wu, R. Li, P. Wang, Hydrophobic light-to-heat conversion membranes with self-healing ability for interfacial solar heating. Adv. Mater. **27**, 4889–4894(2015). <https://doi.org/10.1002/adma.201502362>

[S29] X. Wang, Q. Liu, S. Wu, B. Xu, H. Xu, Multilayer polypyrrole nanosheets with self-organized surface structures for flexible and efficient solar-thermal energy conversion. Adv. Mater. **31**, e1807716(2019). <https://doi.org/10.1002/adma.201807716>

[S30] Y. Shi, R. Li, Y. Jin, S. Zhuo, L. Shi et al., A 3D photothermal structure toward improved energy efficiency in solar steam generation. Joule **2**, 1171–1186(2018). <https://doi.org/10.1016/j.joule.2018.03.013>

[S31] H. Li, H. Wen, Z. Zhang, N. Song, R.T.K. Kwok et al., Reverse thinking of the aggregation-induced emission principle: amplifying molecular motions to boost photothermal efficiency of nanofibers. Angew. Chem. Int. Ed **59**, 20371–20375(2020). <https://doi.org/10.1002/anie.202008292>

[S32] J. Jia, W. Liang, H. Sun, Z. Zhu, C. Wang et al., Fabrication of bilayered attapulgite for solar steam generation with high conversion efficiency. Chem. Eng. J. **361**, 999–1006(2019). <https://doi.org/10.1016/j.cej.2018.12.157>

[S33] C. Song, D. Qi, Y. Han, Y. Xu, H. Xu et al., Volatile-organic-compound-intercepting solar distillation enabled by a photothermal/photocatalytic nanofibrous membrane with dual-scale pores. Environ. Sci. Technol. **54**, 9025–9033(2020). <https://doi.org/10.1021/acs.est.9b07903>

[S34] Y. Xu, J. Ma, Y. Han, H. Xu, Y. Wang et al., A simple and universal strategy to deposit Ag/polypyrrole on various substrates for enhanced interfacial solar evaporation and antibacterial activity. Chem. Eng. J. **384**, 123379(2020). <https://doi.org/10.1016/j.cej.2019.123379>
